# Serinol in the Solid State: Structural Diversity and Packing Motifs in Free Base and Salt Forms

**DOI:** 10.1021/acsomega.6c06041

**Published:** 2026-08-01

**Authors:** Rachele Maschio, Silvia Zampini, Toni Grell, Marco Vandone, Fabio Travagin, Luciano Lattuada, Giovanni B. Giovenzana, Valentina Colombo

**Affiliations:** † Dipartimento di Scienze del Farmaco, Università del Piemonte Orientale, Largo Donegani 2/3, 28100 Novara, Italy; ‡ Dipartimento di Chimica, Università degli Studi di Milano, Via Golgi 19; UdR INSTM, 20133 Milano, Italy; § Bracco Imaging SpA, Via Egidio Folli 50, 20134 Milano, Italy

## Abstract

Serinol (2-amino-1,3-propanediol) is a key aminodiol used on a multiton industrial scale for the synthesis of iopamidol, a widely employed iodinated contrast agent in computed tomography (CT). Despite its small size and structural simplicity, serinol synthesis and purification remain costly and hazardous. Its isolation from process waste streams typically relies on energy-intensive techniques such as high-vacuum distillation or adsorption–desorption on ion exchange resins, followed by evaporation of large water volumes. Crystallization of serinol offers a more sustainable and potentially scalable alternative. However, systematic crystallographic studies aimed at understanding its solid-state behavior remain scarce. In this work, we report a structural investigation of serinol in its free base form and four of its salts: hemioxalate, hydrochloride, stearate, and adamantane-1-carboxylate. The high density of polar protic functional groups in serinol promotes rich supramolecular architectures, governed by extensive and directional hydrogen-bonding networks. The combined spectroscopic and crystallographic analysis supports the formation of ammonium salts and highlights the structural factors governing their solid-state organization and supramolecular assembly. These insights into the solid-state structure of serinol derivatives lay the groundwork for the rational design of crystallization-based purification of serinol in industrial settings.

## Graphical abstract

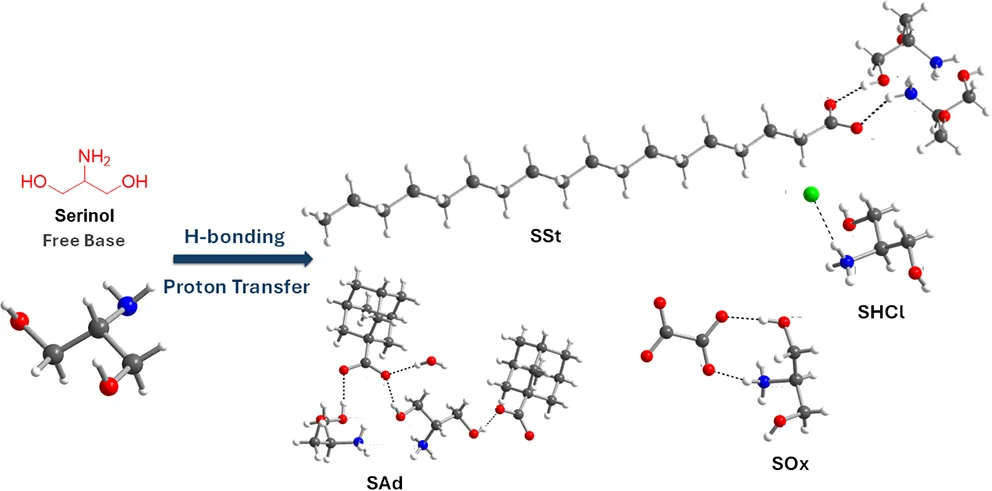

## Introduction

Serinol (2-amino-1,3-propanediol) is a hygroscopic and water-soluble aminodiol that crystallizes as colorless solid with a melting point of 52–56 °C and has a boiling point of 115–116 °C at 0.06 mmHg (Scheme 1).^1,2^ Naturally occurring sources of serinol include sugar cane (*Saccharum officinarum*) infected by *Helminthosporium sacchari*,^3^ and soybean nodules colonized by *Bradyrhizobium spp.*,^4^ where it participates in the biosynthesis of rhizobitoxine.^5^ Among serinol derivatives of biological relevance, sphingosines stand out as key molecules involved in eukaryotic cell growth, apoptosis, and numerous other regulatory processes.^6^ Thanks to its small size and dense functionalization, serinol is a highly versatile building block employed in a wide range of chemical transformations. In the pharmaceutical industry, it serves as the key precursor in the multiton industrial synthesis of iopamidol, an iodinated contrast agent widely used for computed tomography (CT).^7^ Serinol has also been explored for the synthesis of serinol nucleic acids (SNAs),^8^ antiviral compounds,^9^ and antitumor agents.^10^ Beyond medicinal chemistry, serinol finds application in the production of polymers,^11^ surfactants,^12^ and cross-linking agents,^13^ and CO_2_ scavenger^14^ (Figure 1). Several bioactive compounds also feature the serinol substructure, such as fingolimod,^15^ chloramphenicol,^16^ and the wide family of sphingolipids (Figure 1).^17^

**Scheme 1:**
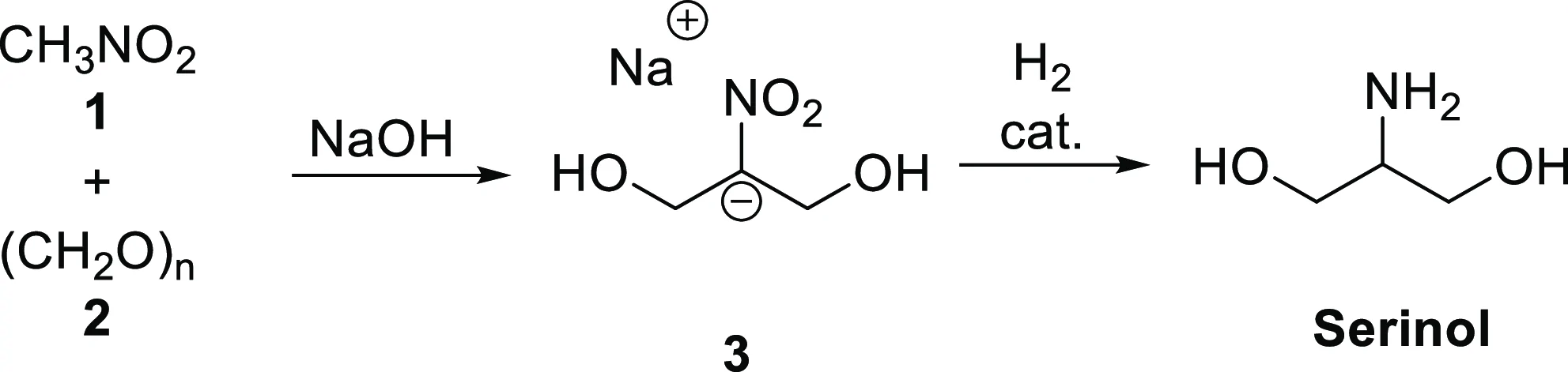
Industrial Synthesis of Serinol from Nitromethane (**1**) and Paraformaldehyde (**2**) through the Sodium Salt of 2-nitro-1,3-propanediol (**3**), Followed by Catalytic Hydrogenation to Serinol

**Figure 1:**
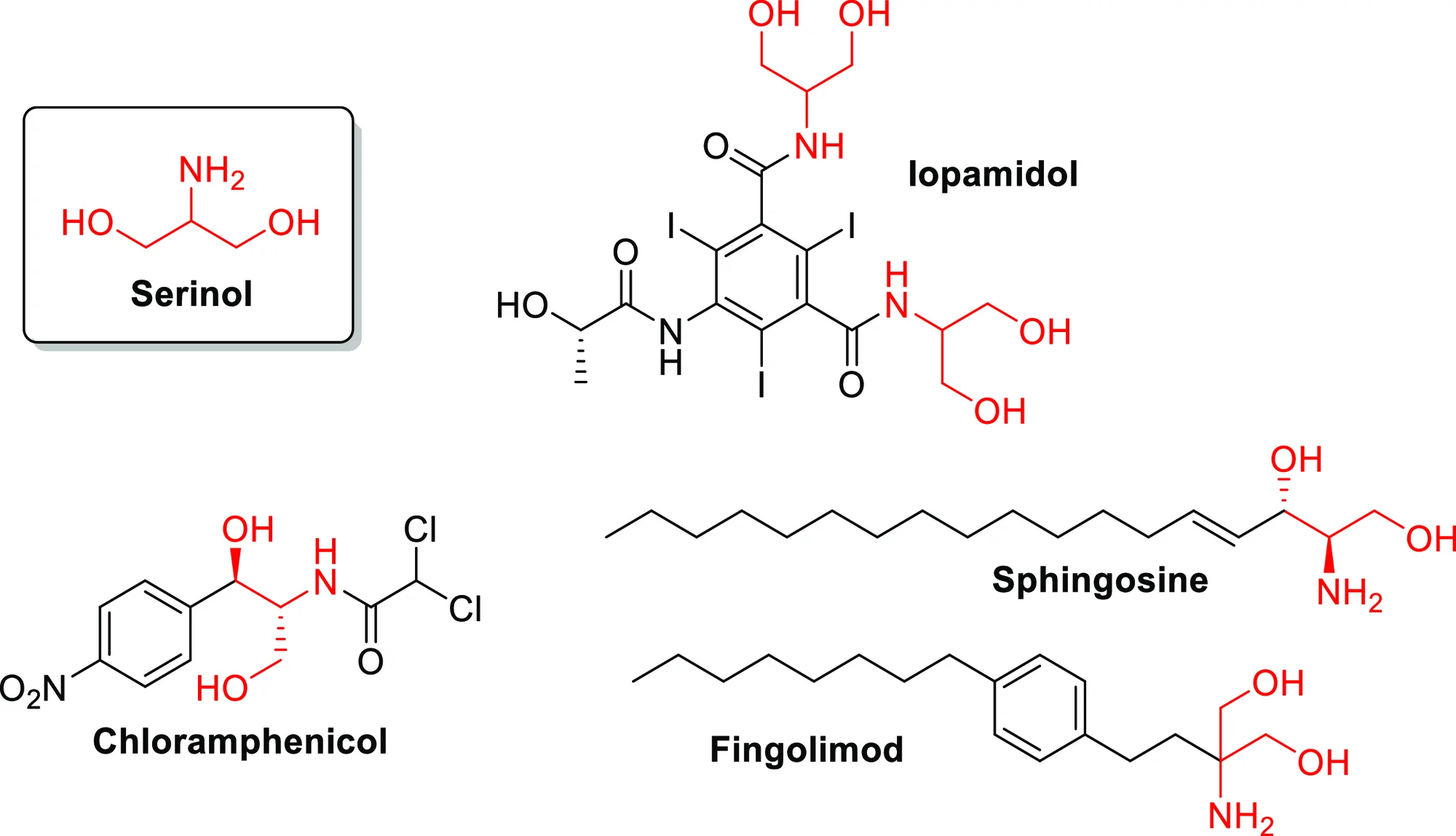
Serinol and some of its derivatives.

The industrial synthesis of serinol involves the Henry reaction of nitromethane (**1**) and paraformaldehyde (**2**) in methanol in the presence of sodium hydroxide, to give the sodium salt of 2-nitro-1,3-propanediol (**3**). The latter is hydrogenated in the presence of Raney-Ni, Rh, Pd or Pt to give serinol.^18^ (Scheme 1).

Owing to the industrial relevance of serinol, different alternative synthetic or biotechnological processes have been studied, such as the catalytic reduction of serine,^19^ of dihydroxyacetone oxime^20^ or imine^21^ and different biotransformations,^22,23^ but to date none of them is currently operating on an industrial scale.

Depending on the specific production process, crude serinol may contain impurities such as aminoalcohols, including tris­(hydroxymethyl)­aminomethane, ethanolamine, *N*-methylserinol, and isoserinol (3-amino-1,2-propanediol). To meet pharmaceutical-grade purity requirements, the removal of these byproducts is essential. Purification is typically achieved through a combination of distillation and crystallization techniques. In addition to ensuring purity, these operations can also be effectively employed for the recovery of serinol from process residues, contributing both to improved sustainability and to the reduction of overall production costs in industrial settings. Distillation of serinol is an energy-intensive process, requiring high temperatures and reduced pressure to be effectively performed. In contrast, crystallization represents a more sustainable and potentially scalable alternative, as it is generally less energy demanding.

Serinol can be crystallized and purified either as the free base or in the form of selected salts, particularly oxalate and hydrochloride, offering viable options for its isolation under milder and more environmentally friendly conditions. Indeed, it is known that the free base of serinol can be obtained through subambient temperature crystallization from lower aliphatic alcohols such as 1-butanol, 2-butanol, and 1-pentanol.^24^ In these conditions, it forms a hygroscopic crystalline solid characterized by a relatively low melting point (lit.^25^ mp 55–58 °C, commercial suppliers report melting points in the range 51–58 °C), both properties negatively affecting its storage and handling. The hydrochloride salt of serinol is also known to be a highly hygroscopic solid (mp 101–103 °C), typically requiring crystallization from anhydrous ethanol under strictly dry conditions,^26^ however, a patent claims its successful crystallization from methanol with no such stringent precautions (lit.^27^ mp 106–108 °C). Serinol can also be precipitated as oxalate salts, though the literature remains ambiguous on the exact stoichiometry of the reported products. In fact, both the 1:1 serinol oxalate (CAS 325469-08-7) and the 2:1 serinol oxalate, commonly referred to as “hemioxalate” (CAS 24070-20-0), have been reported, but only one melting point is documented for the hemioxalate (lit.^28^ mp 202–203 °C).

From the crystallographic point-of-view, only a limited number of crystal structures containing serinol have been reported to date in the Cambridge Structural Database (CSD, Table S1).^29^ Serinol has been employed as a buffering agent, ligand, or counterion in the synthesis of complex polyoxometalates, including Krebs-type sandwich nickel tungstobismuthates,^30^ Anderson–Evans tungstoantimonates,^31^ and hybrid organic–inorganic polyoxotungstates.^32^ Moreover, it has been investigated as a biocompatible salifying agent for selected active pharmaceutical ingredients (APIs), such as tolfenamic acid,^33^ and flufenamic acid,^34^ with the goal of forming supramolecular gels for topical biomedical applications. Despite these examples, a systematic investigation of serinol itself, particularly its salt-forming ability with compounds of potential industrial relevance, has not yet been undertaken. It is in fact interesting to note that the presence of hydrogen bond donor and acceptor groups on each of the three carbon atoms of serinol could enable a rich variety of crystal packing arrangements in the solid crystalline state. Indeed, a detailed understanding of this behavior could guide the rational design of stable crystalline serinol salts, thereby increasing the understanding of and facilitating more efficient recovery and purification processes. Building on these foundations, in this work, we report the synthesis and single-crystal X-ray diffraction characterization of serinol in its free base form (**SFB**) and as four distinct salts: hemioxalate (**SOx**), hydrochloride (**SHCl**), stearate (**SSt**), and adamantane-1-carboxylate (**SAd**). Through a systematic structural comparison, we elucidate how conformational flexibility and hydrogen-bonding capability influence the molecular arrangement and packing motifs across these crystalline forms. This study provides valuable insights into the solid-state behavior of serinol, which may inform future studies aimed at optimizing its purification, handling, and potential large-scale industrial recovery.

## Experimental Section

### Materials and Methods

Solvents and starting materials were purchased from Merck or TCI and used without further purification. All aqueous solutions were prepared from ultrapure laboratory grade water (18MΩ cm) obtained from a Millipore/Milli-Q purification system. IR spectra were acquired over the range of 4000–400 cm^–1^ in attenuated total reflectance (ATR) on a diamond crystal by means of a PerkinElmer Paragon 1000 spectrometer (Figure S6).^1^H and^13^C NMR spectra were recorded on a Bruker Avance Neo 400 spectrometer operating at 9.4 T (Figures S7–S16). Chemical shifts are reported in ppm with the protic impurities of the deuterated solvent as the internal reference. TLC was performed with silica gel (MN Kieselgel 60 F254) and visualized by UV light or sprayed with the Dragendorff reagent or alkaline KMnO_4_. Column chromatography was carried out on Macherey-Nagel Silica gel 60 (0.063–0.200 mm).

### Preparation of Serinol Salts

#### Serinol Free Base (**SFB**)

Crystals of the free base were obtained dissolving serinol (15.2 g) in boiling 2-butanol (30 mL) and letting the solution cool to room temperature and evaporate for 1 week. The mother liquor was discarded, the colorless crystals were washed with cold 2-butanol, collected and dried *in vacuo* at room temperature. M.p.: 51.9–53.0 °C. ^1^H NMR (400.2 MHz, D_2_O, 298 K) δ 3.61 (dd, *J* = 11.3, 5.2 Hz, 2H), 3.50 (dd, *J* = 11.3, 6.3 Hz, 2H), 2.92 (tt, *J* = 6.2, 5.4 Hz 1H); ^13^C NMR (100.6 MHz, D_2_O, 298 K) δ 63.0 [CH_2_], 53.0 [CH]. IR-ATR: 3311 cm^–1^ ν NH_2_ (m), 3180 cm^–1^ ν OH (s), 1604 cm^–1^ δ NH_2_ (m), 1040 cm^–1^ ν C–O (s), 670 cm^–1^ δ NH_2_ (m).

#### Serinol Hemioxalate (**SOx**)

Crystals of the oxalate salt were obtained by dissolving serinol (49.7 mg, 0.545 mmol) and oxalic acid (68.8 mg, 0.545 mmol) in 0.7 mL of water. Methanol (0.7 mL) was added dropwise in 5 min to this solution until crystallization started. The mixture was refluxed for 5 min until a clear solution was obtained, which was stored at 4 °C for 18 h. The mother liquor was discarded, the colorless crystals were washed with methanol, collected and dried. M.p.: 205.5–207.0 °C. ^1^H NMR (400.2 MHz, D_2_O, 298 K) δ 3.82 (dd, *J* = 12.3, 4.5 Hz, 2H), 3.71 (dd, *J* = 12.3, 6.7 Hz, 2H), 3.43 (tt, *J* = 6.7, 4.5 Hz, 1H); ^13^C NMR (100.6 MHz, D_2_O, 298 K) δ 173.3 [C], 58.6 [CH_2_], 54.1 [CH]. IR-ATR: 3240 cm^–1^ ν OH (s), 3000 cm^–1^ ν NH_3_^+^ (m), 1610 cm^–1^ 1380 cm^–1^ ν COO^–^ (s), 1570 cm^–1^ 1523 cm^–1^ δ NH_3_^+^ (s), 1040 cm^–1^ ν C–O (s).

#### Serinol Hydrochloride (**SHCl**)

Crystals of the hydrochloride salt were obtained by dissolving serinol (91.1 mg, 1.00 mmol) and hydrochloric acid (37% w/w, 98.5 mg, 1.00 mmol) in 0.5 mL of ethanol. The mixture was refluxed until a clear solution was obtained, which was stored at 4 °C for 72 h. The mother liquor was discarded, the colorless crystals were washed with ethanol, collected and dried *in vacuo* at room temperature. M.p.: 99.1–102.0 °C. ^1^H NMR (400.2 MHz, D_2_O, 298 K) δ 3.84 (dd, *J* = 12.3, 4.4 Hz, 2H), 3.73 (dd, *J* = 12.3, 6.7 Hz, 2H), 3.45 (tt, *J* = 6.2, 4.5 Hz, 1H); ^13^C NMR (100.6 MHz, D_2_O, 298 K) δ 58.6 [CH_2_], 54.1 [CH]. IR-ATR: 3300 cm^–1^ ν OH (s), 3000 cm^–1^ 2957 cm^–1^ ν NH_3_^+^ (m), 1616 cm^–1^ 1572 cm^–1^ δ NH_3_^+^ (s), 1035 cm^–1^ ν C–O (s).

#### Serinol Stearate (**SSt**)

Crystals of the stearate salt were obtained by dissolving serinol (45.6 mg, 0.500 mmol) and stearic acid (142 mg, 0.500 mmol) in 8 mL of methanol. The mixture was refluxed, and a clear solution was obtained. The solution was stored at room temperature for 8 h and at 4 °C for 24 h. The mother liquor was discarded, the colorless crystals were washed with methanol, collected and dried *in vacuo* at room temperature. M.p.: 102.8–105.1 °C. ^1^H NMR (400.2 MHz, CD_3_OD, 303 K) δ 3.73 (dd, *J* = 11.5, 4.6 Hz, 2H), 3.63 (dd, *J* = 11.5, 6.6 Hz, 2H), 3.19 (tt, *J* = 6.6, 4.6 Hz, 1H), 2.17 (t, *J* = 7.6 Hz, 2H), 1.59 (q, *J* = 7.2 Hz, 2H), 1.34–1.29 (m, 28H), 0.90 (bt, *J* ≈ 6.9 Hz, 3H); ^13^C NMR (100.6 MHz, CD_3_OD, 303 K) δ 182.4 [C], 60.8 [CH_2_], 55.9 [CH], 38.7 [CH_2_], 33.1 [CH_2_], 30.78 [8 CH_2_], 30.75 [CH_2_], 30.73 [CH_2_], 30.6 [CH_2_], 30.5 [CH_2_], 27.5 [2 CH_2_], 23.7 [CH_2_], 14.4 [CH_3_]. IR-ATR: 3151 cm^–1^ ν OH (s), 2914 cm^–1^ 2850 cm^–1^ ν NH_3_^+^ (m), 1540 cm^–1^ 1408 cm^–1^ ν COO^–^ and δ NH_3_^+^(s), 1076 cm^–1^ ν C–O (s), 1644 cm^–1^ δ NH_3_^+^/H-bonded OH bending (s).

#### Serinol Adamantane-1-carboxylate (**SAd**)

Crystals of the adamantane-1-carboxylate salt were obtained by dissolving serinol (91.1 mg, 1.00 mmol) and adamantane-1-carboxylic acid (180 mg, 1.00 mmol) in 3 mL of methanol. The mixture was heated under reflux. The clear solution obtained was stored at 4 °C for 1 week. The mother liquor was discarded, the colorless crystals were washed with cold methanol, collected and dried *in vacuo* at room temperature. M.p.: 157.9–162.1 °C. ^1^H NMR (400.2 MHz, CD_3_OD, 303 K) δ 3.73 (dd, *J* = 11.6, 4.7 Hz, 2H), 3.63 (dd, *J* = 11.5, 6.5 Hz, 2H), 3.18 (tt, *J* = 6.5, 4.7 Hz, 1H), 1.96 (m, 3H), 1.88 (m, 6H), 1.73 (m, 6H);^13^C NMR (100.6 MHz, CD_3_OD, 303 K) δ 186.2 [C], 61.1 [CH_2_], 55.9 [CH], 42.9 [C], 41.0 [CH_2_], 38.0 [CH_2_], 30.0 [CH]. IR-ATR: 3395 cm^–1^ 3334 cm^–1^ ν NH_2_ (m) and ν OH­(H_2_O) (b), 3073 ν OH (s), 2900 cm^–1^ 2845 cm^–1^ ν NH_3_^+^ (m), 1645 ν COOH (m), 1602 cm^–1^ δ NH_3_^+^ (s), 1540 cm^–1^ 1390 cm^–1^ ν COO^–^ (s), 1070 cm^–1^ ν C–O (s).

#### Single-Crystal X-ray Diffraction (SC-XRD)

SC-XRD data were collected with an XtaLAB Synergy diffractometer (RIGAKU). The radiation source was a copper microfocus sealed X-ray tube anode (Cu–Kα, λ = 1.54184 Å) with the generator working at 50 kV and 1 mA. The data reduction was carried out with CrysAlis Pro^35^ version 1.171.42.90a using an empirical absorption correction with spherical harmonics (SCALE3 ABSPACK). The structures were solved by dual space methods with SHELXT-2017 ^36^ and refined with SHELXL-2019^36^ using the WinGX program suite.^37^ Structure refinement was done using full-matrix least-squares routines against F^2^. All hydrogen atoms on carbon were calculated on idealized positions. Hydrogen atoms connected to heteroatoms were located as residual electron density peaks. Their positions were refined using a riding model. CCDC 2473051–55 contain the supplementary crystallographic data for this paper. These data and additional information can be obtained free of charge via https://summary.ccdc.cam.ac.uk/structure-summary-form (or from the Cambridge Crystallographic Data Centre, 12 Union Road, Cambridge CB2 1EZ, UK; fax: (+44)­1223–336–033; or deposit@ccdc.cam.uk).

## Results and Discussion

Crystallization experiments were carried out to obtain single crystals suitable for X-ray diffraction and to determine the crystal structures of serinol free base and the newly identified serinol salts. Despite numerous attempts using different crystallization techniques, single crystals could be obtained only by a slow cooling method. In this approach, serinol and the corresponding cocrystallizing agent were refluxed in methanol, ethanol, or a water–methanol mixture, and the resulting solution was stored at 4 °C to allow for gradual crystal growth. The crystal structures of serinol free base (**SFB**) and the 4 salts (**SOx**, **SHCl**, **SSt**, and **SAd**) were indeed determined by single crystal X-ray diffraction (SC-XRD).

In all the compounds, the crystal packing is governed by extensive hydrogen bonding, enabled by the presence of two hydroxyl groups and one amino group on the serinol molecule. Notably, the available evidence suggests that in all salt forms investigated, the amino group is likely protonated, contributing to the formation of robust, directional supramolecular architectures stabilized by multiple hydrogen bond interactions. This protonation behavior is consistent with the significant difference in p*K*_a_ values between the amino group of serinol (p*K*_a_ ≈ 12.2) and the acids employed as coformers – hydrochloric acid (p*K*_a_ ≈ −7), oxalic acid (p*K*_a1_ ≈ 1.25, p*K*_a2_ ≈ 4.26), stearic acid (p*K*_a_ ≈ 4.75), and adamantane-1-carboxylic acid (p*K*_a_ ≈ 4.9). The marked acidity of these coformers renders proton transfer to serinol thermodynamically favorable in all cases, thereby rationalizing the consistent protonation of the amino group across the series. This interpretation is also consistent with the commonly used Δp*K*_a_ criterion for acid–base multicomponent crystals, according to which large positive Δp*K*_a_ values generally favor salt formation, although the final ionization state may also be influenced by the crystalline environment.^38^

Further experimental evidence supports the occurrence of proton transfer in the investigated systems. The ^1^H NMR spectra of the isolated compounds show systematic downfield shifts of the protons located α and β to the amino group relative to the free base. Protonation of amines is known to induce significant deshielding effects, particularly for α-CH protons (typically 0.4–0.6 ppm) and, to a lesser extent, for β-CH_2_ protons, as documented in classical NMR studies of amine protonation.^39^ In the present series, the α-CH signal shifts from +0.26 to +0.53 ppm relative to the free base, while the β-CH_2_ signals shift from +0.12 to +0.23 ppm (Table 1). These values are consistent with protonation of the amine group species in solution.

**Table 1:** p*K*_a_ Values, ^1^H NMR Chemical Shift Variations (Δδ) and Selected Bond Distances from SC-XRD

compound	p*K*_a_	Δδ α-CH (ppm)	Δδ β-CH_2_ (ppm)	C–N^a^ (Å)	C–O1 (Å)	C–O2 (Å)	Δ(C–O) (Å)
**SFB**	12.2	0.00	0.00, 0.00	[A] 1.4696(17) [B] 1.4712(18)	–	–	–
**SHCl**	–7	+0.53	+0.22, +0.23	1.5176(12)	–	–	–
**SOx**	1.25, 4.26	+0.51	+0.21, +0.21	1.4967(8)	1.2468(8)	1.2698(8)	0.023
**SSt**	4.75	+0.27	+0.12, +0.13	1.492(6)	1.262(5)	1.271(6)	0.009
**SAd**	4.9	+0.26	+0.12, +0.13	[A] 1.500(4) [B] 1.493(4)	[A] 1.256(3) [B] 1.250(3)	[A] 1.282(3) [B] 1.267(3)	[A] 0.026 [B] 0.017

a Δδ values are calculated relative to **SFB**. For **SFB**, the reported p*K*_a_ refers to the conjugate acid of serinol; for all other entries, p*K*_a_ values refer to the corresponding acid coformer. A and B denote crystallographically independent molecules/groups.

Additional support is provided by the solid-state structural data. In all organic derivatives (**SOx**, **SSt** and **SAd**), the C–O bond distances within the carboxylate groups are nearly equal, which is characteristic of deprotonated carboxylate anions. The small Δ­(C–O) values observed (Table 1) are consistent with charge delocalization within the COO^–^ group. Moreover, the hydrogen-bonding patterns observed in the crystal structures are in agreement with charge-assisted N–H···O interactions typically found in ammonium carboxylate salts, a robust supramolecular synthon widely exploited in layered organic crystals.^40^

It should be noted, however, that conventional single-crystal X-ray diffraction does not allow an unambiguous localization of hydrogen atoms involved in acid–base equilibria. Therefore, while the combined spectroscopic and crystallographic evidence strongly supports the formulation of these compounds as ammonium salts, the exact position of the proton cannot be definitively established in all cases.

Table 2 contains the summary of the crystallographic data for each crystal structure. A complete list of the hydrogen bond interactions for each structure is reported in the Supporting Information (Tables S2 and S3).

**Table 2:** Summary of Crystallographic Data

compound	**SFB**	**SOx**	**SHCl**	**SSt**	**SAd**
formula	C_3_H_9_NO_2_	C_4_H_10_NO_4_	C_3_H_10_ClNO_2_	C_21_H_45_O_4_N	C_28_H_52_N_2_O_9_
FW	91.11	136.13	127.57	375.58	560.71
crystal system	orthorhombic	monoclinic	monoclinic	triclinic	monoclinic
space group	*Pna*2_1_	*C*2/*c*	*P*2_1_/*n*	*P*1̅	*P*2_1_/*c*
a (Å)	22.2516(3)	11.4891(4)	10.9467(4)	5.4799(9)	18.4956(5)
b (Å)	7.19260(10)	5.2314(2)	5.4629(2)	7.7634(8)	6.5953(2)
c (Å)	5.83480(10)	20.7377(7)	11.0792(4)	27.3750(15)	24.1026(8)
*α* (°)	90	90	90	93.936(7)	90
*β* (°)	90	95.811(3)	110.015(4)	92.738(11)	99.908(3)
*γ* (°)	90	90	90	97.619(12)	90
V (Å^3^)	933.84(2)	1240.02(8)	622.53(4)	1149.7(2)	2896.28(15)
Z	8	8	4	2	4
temperature (K)	150(2)	298(2)	298(2)	150(2)	150(2)
ρ_(calcd)_ (g·cm^‑3^)	1.296	1.458	1.361	1.085	1.286
μ (mm^–1^)	0.905	0.130	0.516	0.575	0.778
F(000)	400.0	584.0	272.0	420.0	1224.0
crystal size (mm^3^)	0.18 × 0.16 × 0.11	0.25 × 0.2 × 0.18	0.4 × 0.32 × 0.21	0.08 × 0.05 × 0.04	0.08 × 0.05 × 0.04
Θ range (°)	7.946 to 160.286	3.948 to 62.744	4.516 to 62.22	6.484 to 136.46	7.446 to 126.86
limiting indices	–28 ≤ *h* ≤ 24–9 ≤ *k* ≤ 9–7 ≤ *l* ≤ 6	–16 ≤ *h* ≤ 16–7 ≤ *k* ≤ 7–29 ≤ *l* ≤ 29	–15 ≤ *h* ≤ 15–7 ≤ *k* ≤ 7–15 ≤ *l* ≤ 15	–6 ≤ *h* ≤ 5–9 ≤ *k* ≤ 9–32 ≤ *l* ≤ 32	–21 ≤ *h* ≤ 20–6 ≤ *k* ≤ 7–27 ≤ *l* ≤ 27
reflns collected/unique	3585/1479 [R_int_ = 0.0123]	6592/1909 [R_int_ = 0.0048]	13364/1894 [R_int_ = 0.0469]	12233/4194 [R_int_ = 0.0871]	21738/4610 [R_int_ = 0.0529]
data/restraints/param	1479/1/141	1909/3/95	1894/0/84	4194/6/256	4610/10/382
completeness (%) to θ (°)	99.9 (67.7)	100 (25.2)	99.8 (25.2)	99.5 (67.7)	97.2 (63.4)
max. and min transmission	1.00000 and 0.47798	1.00000 and 0.88840	1.00000 and 0.72338	1.00000 and 0.45265	1.00000 and 0.76806
final *R* indices [I > 2σ(I)]	R_1_ = 0.0283, w*R*_2_ = 0.0783	R_1_ = 0.0279, w*R*_2_ = 0.0771	R_1_ = 0.0350, w*R*_2_ = 0.0937	R_1_ = 0.1274, w*R*_2_ = 0.3727	R_1_ = 0.0630, w*R*_2_ = 0.1766
*R* indices (all data)	R_1_ = 0.0285, w*R*_2_ = 0.0786	R_1_ = 0.0289, w*R*_2_ = 0.0780	R_1_ = 0.0356, w*R*_2_ = 0.0945	R_1_ = 0.1764, w*R*_2_ = 0.4220	R_1_ = 0.0824, w*R*_2_ = 0.1903
GooF on F^2^	1.006	1.059	1.099	1.446	1.045
largest diff peak and hole (e·Å^–3^)	0.23 and −0.18	0.42 and −0.20	0.33 and −0.41	0.44 and −0.53	0.43 and −0.28
CCDC	2473055	2473051	2473052	2473054	2473053

### Crystal Structure Analysis

#### Serinol Free Base (**SFB**)

Serinol in its free base form crystallizes in the orthorhombic space group *Pna*2_1_, with two independent molecules in the asymmetric unit (*Z* = 8, *Z’* = 2, Wyckoff sites *a*, Figures 2a and S1). The two serinol molecules interact via an intermolecular hydrogen bond [O1B–H···O2A = 2.6601(17) Å]. Both O1B and O2A participate in additional hydrogen bonds: O1B accepts a hydrogen bond from the hydroxyl group of a symmetry-related molecule [O2B*–H···O1B = 2.7146(16) Å; *symmetry code:* 1–*x*, 1–*y*, 1/2 *+ z*], while O2A donates a hydrogen bond to an amino group [O2A–H···N1B* = 2.7321(17) Å; *symmetry code:* 1–*x*, 2–*y, −*1/2 *+ z*]. Each functional group of the serinol molecules acts as a hydrogen bond donor or acceptor, creating a dense three-dimensional network. In particular, O1A forms hydrogen bonds with three distinct amino groups, while O2B is involved in hydrogen bonding with two amino groups and one hydroxyl group. The amino groups N1A and N1B each engage in three hydrogen bonds with hydroxyl donors from neighboring molecules. As a result, both independent molecules in the asymmetric unit are linked through hydrogen bonding to six additional serinol molecules in the extended structure.

**Figure 2:**
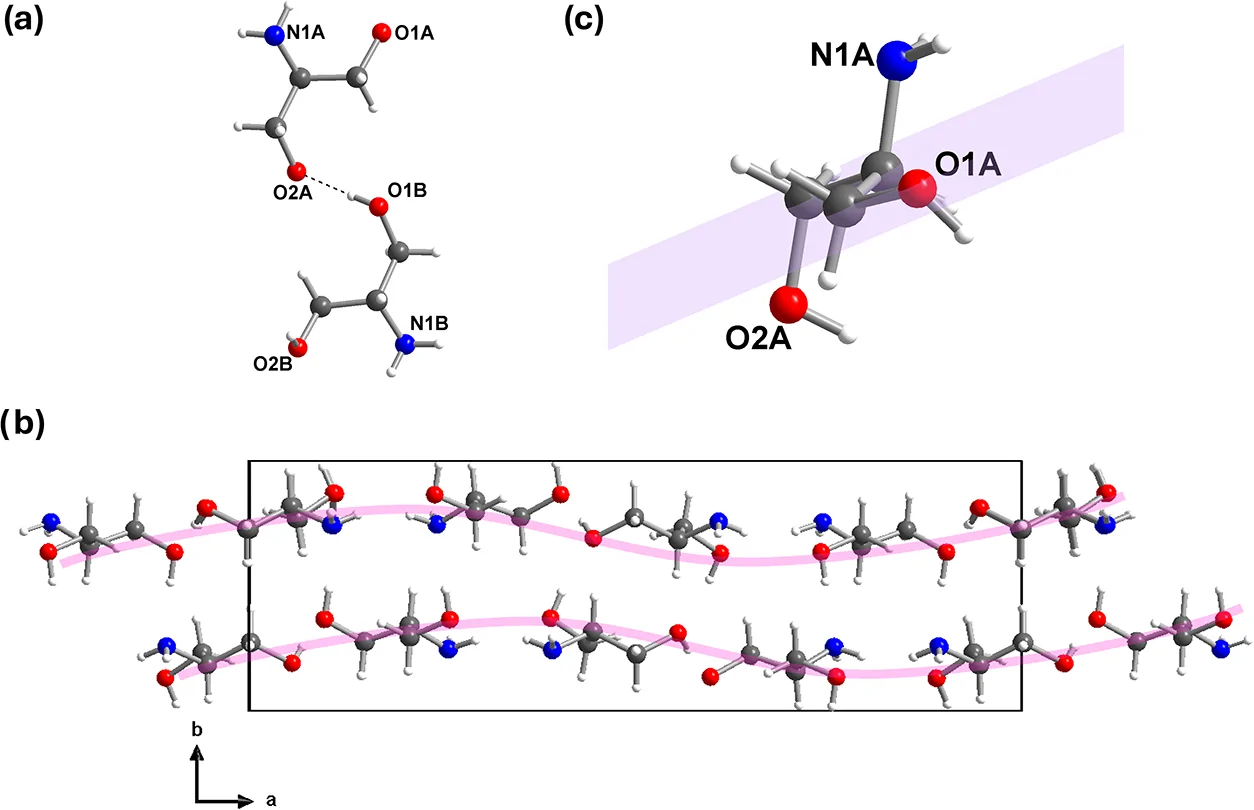
(a) Asymmetric unit of serinol free base (**SFB**), highlighting the intermolecular hydrogen bond O1B–H···O2A [2.6601(17) Å]. (b) View of the crystal packing down to the *c* axis highlighting the wave-like hydrogen-bonding motif propagating along the crystallographic [100] direction. (c) Molecular conformation of both independent serinol molecules in the SFB crystal structure. Color code: C, gray; N, blue; O, red; H, white. Hydrogen bonds are depicted as dashed black lines.

In the three-dimensional arrangement, the crystal structure reveals an undulating, wave-like motif propagating along the [100] direction (Figure 2b). This supramolecular organization might arise from the spatial disposition required to maximize intermolecular hydrogen bonding while minimizing steric repulsion. The two independent serinol molecules adopt identical conformations, as illustrated in Figure 2c. When defining a reference plane through the three carbon atoms, one hydroxyl group lies approximately within the plane, while the other is rotated outward, positioned on the opposite side relative to the amino group.

#### Serinol Hemioxalate (**SOx**)

**SOx** crystallizes in the monoclinic *C2*/*c* space group. The asymmetric unit contains one serinol molecule in a general position (*Z* = 8; *Z*′ = 1, Wyckoff site *f*,) and half of an oxalate anion, which lies on an inversion center (Wyckoff site *b*; multiplicity 4; coordinates: 0, 1/2, 0). As a result, the crystal structure corresponds to a 1:0.5 stoichiometry of serinol to oxalate, defining it as a serinol hemioxalate salt (Figures 3a and S2). The oxalate anion engages in hydrogen bonding with six surrounding serinol molecules. Specifically, one of the oxygen atoms of the oxalate anion (O3) participates in HB interactions with three different serinol molecules. It accepts a hydrogen bond from the O2 hydroxyl group of one molecule [O2–H···O3 = 2.6264(7) Å] and, in addition, forms hydrogen bonds with the amino groups of two further serinol molecules. The other oxygen atom of the oxalate (O4) is also involved in three hydrogen bonds with amino groups of three different molecules. Each serinol molecule donates hydrogen bonds to three oxalate anions through its protonated amino group and one hydroxyl group. It also forms hydrogen bonds with two additional serinol molecules via hydroxyl···hydroxyl interactions [O1–H···O2* = 2.6867(7) Å; *symmetry code:* *–1/2 + *x*, 1/2 + *y*, *z*].

**Figure 3:**
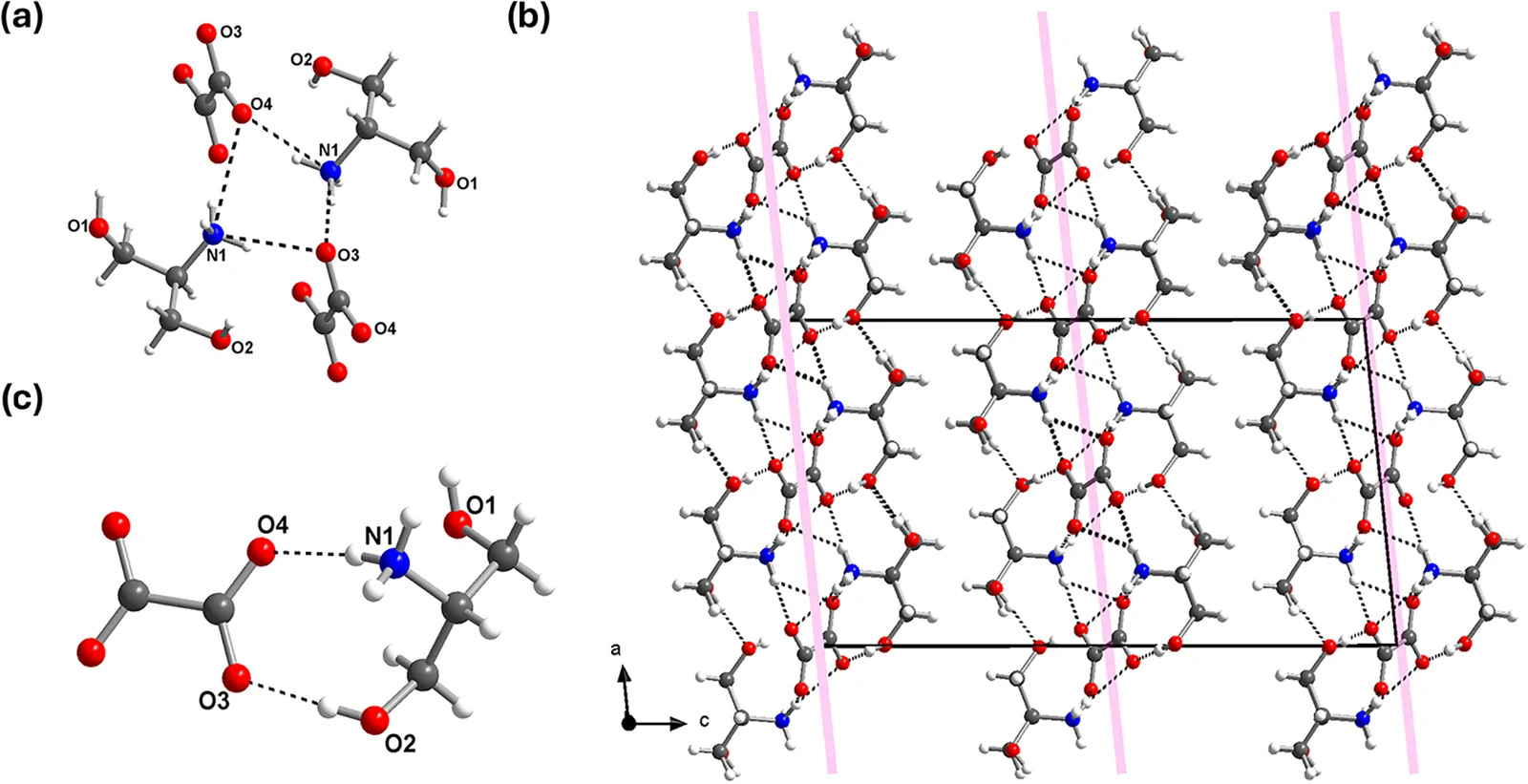
(a) Four-membered hydrogen-bond motif formed between two serinol molecules and two oxalate anions. Selected hydrogen bond distances: N1­(−H)···O3 = 2.8371(7) Å; N1­(−H)···O4 = 3.1025(8) Å. (b) Hydrogen-bonded chains running along the [100] crystallographic direction, viewed along the *b* crystallographic axis. Oxalate anions (highlighted with pink vertical lines) are centrally positioned between serinol molecules and mediate the chain assembly. (c) Close-up of the interactions between a serinol molecule and the oxalate anion. Selected hydrogen bond distances: N1­(−H)···O4 = 3.1025(8) Å; O2­(−H)···O3 = 2.6264(7) Å. Color code: C, gray; N, blue; O, red; H, white. Hydrogen bonds are depicted as dashed black lines.

Notably, the interaction between the protonated amino group (N1) of the serinol molecule and oxalate oxygen atoms generates four-membered hydrogen-bonded ring motifs (Figure 3a). These interactions result in the formation of chains of alternating serinol molecules and oxalate anions running along the [010] direction, which are further connected via hydrogen bonding along [100], with the oxalate anion situated on the axis of the network (Figure 3b).

The conformation of the serinol molecule in this structure is dictated by its hydrogen-bonding interactions. Unlike the extended conformation observed in **SFB**, the two hydroxyl groups and the amino group are oriented in the same direction relative to the carbon backbone, effectively facing the oxalate anion and enabling chain formation (Figure 3c).

#### Serinol Hydrochloride (**SHCl**)

Serinol Hydrochloride (**SHCl**) crystallizes in the *P*2_1_/*c* space group of the monoclinic system with one serinol and one chloride anion that lie in general position (*Z* = 4, *Z’* = 1, Wyckoff site *e* Figures 4 and S3). The unit cell indeed contains four formula units. The chloride ion, acting as a hydrogen bond acceptor, engages in four hydrogen bonds with surrounding serinol molecules: two with protonated amino groups [N1­(−H)···Cl1 = 3.2634(8) Å and N1*­(−H)···Cl1 = 3.2935(9) Å, * = *x*; 1+*y*; *z*] and two with hydroxyl groups [O1*–H···Cl1 = 3.2204(9) Å, *symmetry code:* * = 1/2 – *x*; 1/2 + *y*; 3/2 – *z*; O2*–H···Cl1 = 3.2106(8) Å, *symmetry code:* * = −1/2 + *x*; 1/2 – *y*; −1/2 + *z*]. The protonated amino group points toward the chloride anion, facilitating intermolecular interactions that stabilize the crystalline packing (Figure 4a). Each serinol molecule also forms an intermolecular hydrogen bond with two other serinol molecules via one hydroxyl group and the protonated amino group, acting in both ways as HB donor and acceptor [N1­(−H)···O2* = 2.9353(11) Å, *symmetry code:* * = 1/2 – *x*; – 1/2 + *y*; 5/2 – *z*] (Figure 4b).

**Figure 4:**
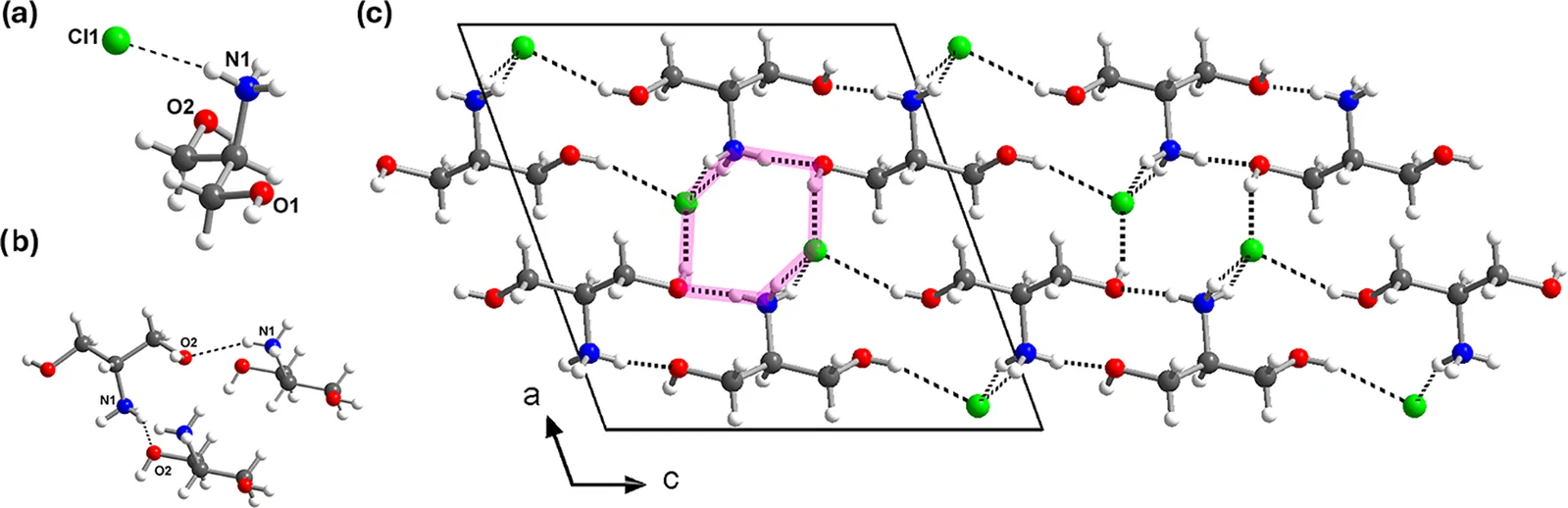
(a) Hydrogen bond interaction between the protonated amino group and a chloride anion. Selected distance: N1­(−H)···Cl1 = 3.2935(9) Å. (b) Hydrogen bonding between three serinol molecules related by a *C*2 screw axis. Selected distance: N1­(−H)···O2 = 2.9353(11) Å. (c) Crystal packing viewed down the *b* axis, highlighting the formation of six-membered hydrogen-bonded rings composed of two serinol molecules and two chloride anions. The HB 6-membered ring is highlighted in pink. Color code: C, gray; N, blue; O, red; H, white; Cl, green. Hydrogen bonds are depicted as dashed black lines.

In the structure, it is interesting to note that two serinol molecules and two chloride anions alternate along the [001] direction, forming a six-membered hydrogen-bonded ring. This motif involves one chloride anion, the protonated amino group of one serinol molecule, and the hydroxyl group of a second serinol molecule, repeated twice in an alternating, clockwise fashion (Figure 4c). This hydrogen-bonding arrangement constrains the serinol molecule into a conformation where the two oxygen atoms lie nearly coplanar with the carbon backbone, and the amino group which is found perpendicular to this plane, stabilizing the overall packing.

#### Serinol Stearate (**SSt**)

Serinol stearate crystallizes in the triclinic space group *P*1^–^. The asymmetric unit contains one serinol molecule, consistent with a protonated form, and one stearate anion, both located in general positions (*Z* = 2; *Z’* = 1; Wyckoff site *a*). These two components are connected through a hydrogen bond between one hydroxyl group of serinol and a carboxylate oxygen atom of the stearate anion (O2­(−H)···O3 = 2.602(4) Å) (Figures 5a and S4). In the crystal structure, each serinol molecule forms interactions with another serinol molecule via reciprocal hydrogen bonds between the protonated amino group and hydroxyl groups, resulting in zigzag chains of serinol units propagating along the *b* axis. These symmetric interactions involve two distinct hydrogen bonds: N1­(−H)···O1* = 2.741(5) Å (symmetry code: * = −*x*, −1–*y*, −*z*) and N1­(−H)···O2* = 2.791(5) Å (symmetry code: * = −*x*, −*y*, −*z*).

**Figure 5:**
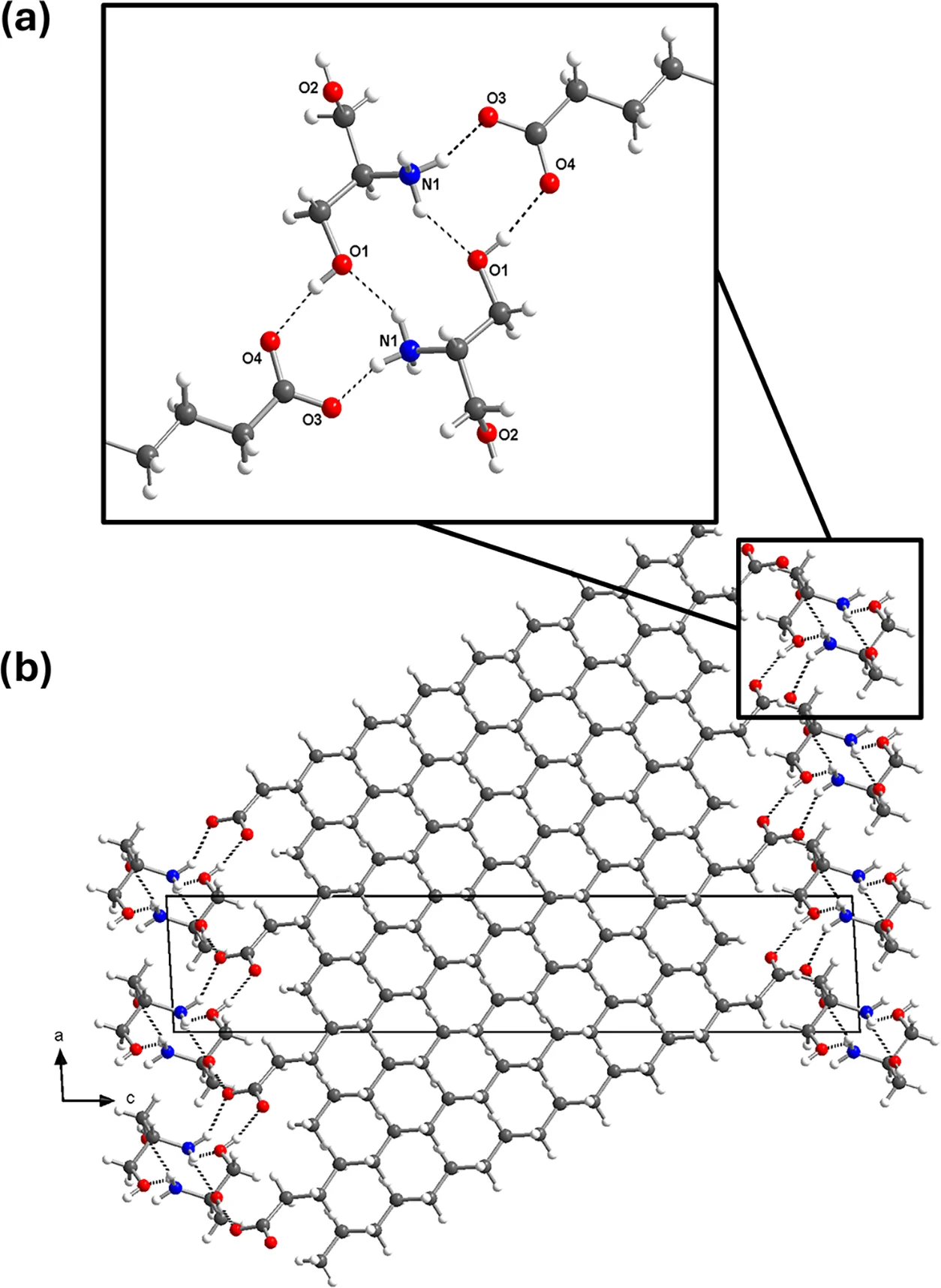
(a) Molecular arrangement of serinol molecules and stearate anion. Selected hydrogen bond distances: N1­(−H)···O3 = 2.701(5) Å; O1­(−H)···O4 = 2.646(5) Å. (b) Representation of the alternation of serinol molecules and stearate anions in the crystal packing. Hydrogen bonds are depicted with dashed black lines. Color code: C, gray; N, blue; O, red; H, white. Hydrogen bonds are depicted as dashed black lines.

Additionally, each serinol molecule engages in hydrogen bonding with two adjacent stearate anions, leading to the formation of alternating layers of serinol chains and stearate molecules perpendicular to the [001] direction. Specifically, each stearate anion interacts with two serinol molecules via both a hydroxyl group and the protonated amino group, through the following hydrogen bonds: O1*–H···O4 = 2.646(5) Å (symmetry code: * = −1 + *x*, 1 + *y*, *z*) and N1*–H···O3 = 2.701(5) Å (symmetry code: * = −1 – *x*, −*y*, −*z*). To accommodate this extensive hydrogen bonding network, the serinol molecule adopts a conformation in which one hydroxyl group is found in the molecular plane defined by the carbon backbone and the other two functional groups (one hydroxyl and one protonated amino groups) are oriented toward the carboxylic group of the stearate anion, forming hydrogen bonds with it. The stearate anion adopts a nearly linear conformation, which facilitates the formation of the well-ordered layers perpendicular to the *c* axis. Overall, the crystal packing is characterized by stacking of serinol cations and stearate anions along the [010] direction, side-by-side alignment along [100], and hydrogen bonding connections with alternating layers of serinol and stearate anion along [001], yielding a three-dimensional supramolecular network (Figure 5b). The structural features observed for **SSt** are in close agreement with those reported for ammonium salts of long-chain fatty acids, which typically adopt lamellar arrangements characterized by the segregation of polar ammonium–carboxylate layers and hydrophobic alkyl chains.^41–43^ In such systems, hydrogen-bonding interactions between ammonium cations and carboxylate anions form extended two-dimensional networks, while the long aliphatic chains promote layered packing dominated by van der Waals interactions.

It is also well documented that ammonium salts of long-chain fatty acids often exhibit limited crystallinity and challenging diffraction behavior, particularly for stearate derivatives, for which difficulties in obtaining high-quality single-crystal data have been reported. The structural characteristics and relatively high residual factors observed for **SSt** are therefore consistent with the intrinsic features of this class of materials. In addition, the relatively low calculated density of **SSt** (1.085 g cm^–3^), significantly lower than those observed for the other salts in this series, is in line with the packing characteristics of long-chain fatty acid salts, where the dominance of extended hydrocarbon chains leads to less efficient packing and increased structural anisotropy. Such density values are commonly associated with layered structures in which densely hydrogen-bonded polar regions alternate with more loosely packed hydrophobic domains.

#### Serinol Adamantane-1-carboxylate (**SAd**)

**SAd** crystallizes in the monoclinic space group *P*2_1_/*c* as a hydrated salt. The asymmetric unit contains two serinol molecules, two adamantane-1-carboxylate anions (*Ad* molecule A and B in the following), and one water molecule, all in general position leading to an hemihydrate salt with formula C_3_H_10_NO_2_·C_11_H_15_O_2_·0.5 H_2_O (*Z* = 4, *Z*′ = 1, Wyckoff site *e*, Figures 6a and S5).

**Figure 6:**
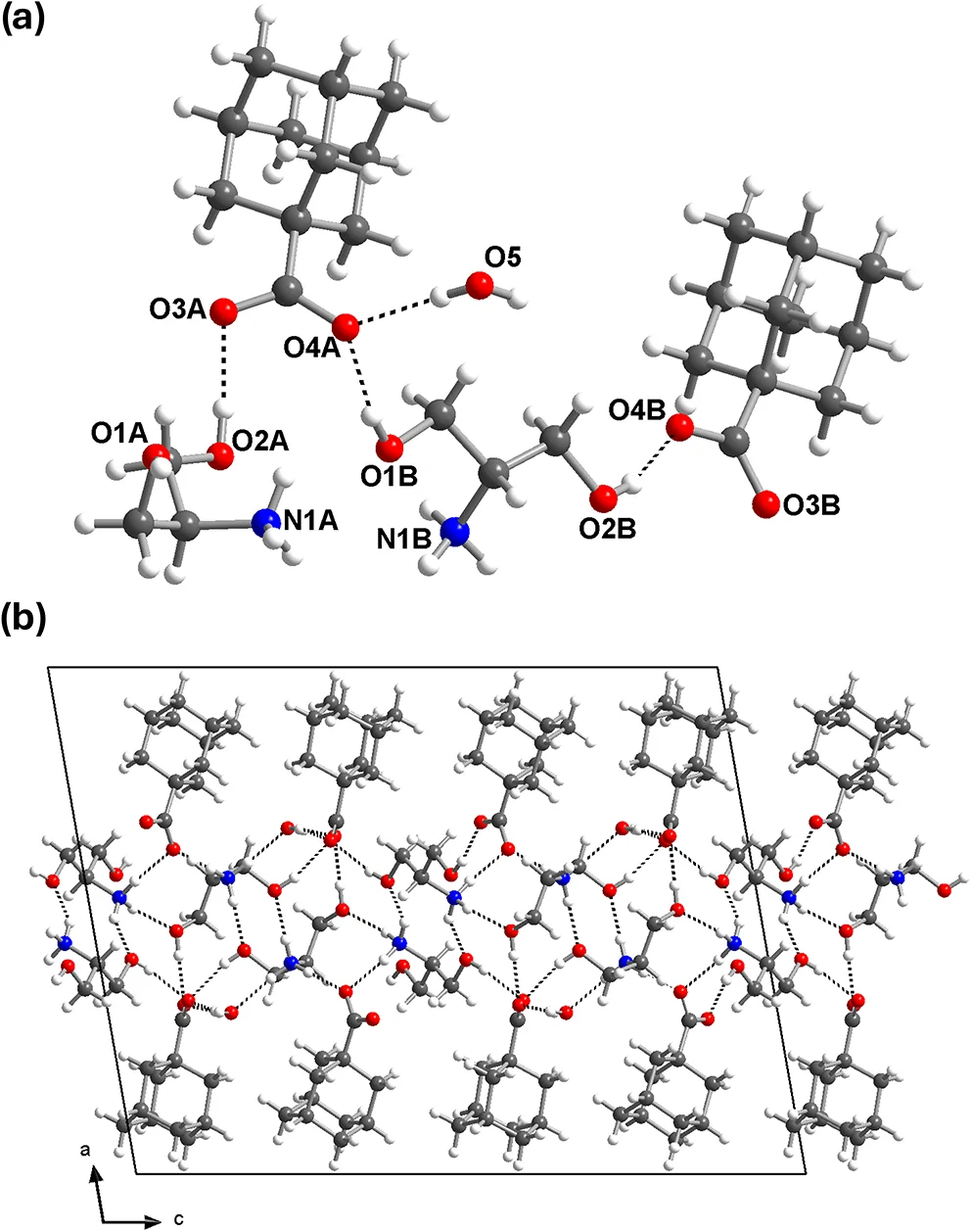
(a) Asymmetric unit of serinol adamantane-1-carboxylate (**SAd**), showing two serinol molecules, two adamantane-1-carboxylate anions, and one water molecule. Selected hydrogen bond distances: O2­(−H)···O7 = 2.675(3) Å, O3­(−H)···O8 = 2.754(3) Å, O9­(−H)···O8 = 2.745(3) Å, O4­(−H)···O6 = 2.615(3) Å. (b) Crystal packing diagram viewed down the *b* axis, showing the formation of chains of serinol, water, and adamantane molecules propagating along the [001] direction. Color code: C, gray; N, blue; O, red; H, white. Hydrogen bonds are shown as dashed black lines.

The water molecule acts as a hydrogen bond acceptor from one serinol molecule and as a donor to two symmetry related adamantane-1-carboxylate anions (*Ad* molecule A in the a.u.), each of which is further engaged in hydrogen bonding with three distinct serinol molecules. The second adamantane anion present in the asymmetric unit (*Ad* molecule B) forms hydrogen bonds with only three serinol molecules. However, a longer intermolecular contact of the type C7B–H···O5­(water) is also observed, with a distance of 3.674(5) Å, suggesting a weak interaction involving the cocrystallized water molecule. The two serinol molecules participate in multiple hydrogen bonding interactions involving three adamantane anions, other serinol molecules, and, in one case, also the water molecule. Hydrogen bond lengths fall all within the range of 2.675(3) to 2.887(3) Å. In the crystal packing, the serinol and water molecules form chains propagating along the [001] direction and aligning side by side along [100] (Figure 6b).

Notably, the two serinol molecules in the asymmetric unit adopt different conformations. One exhibits a relatively planar geometry, with both hydroxyl groups approximately lying in the plane defined by the carbon backbone, while the protonated amino group points above this plane, forming a tetrahedral angle of approximately 111° (Figure 6a, atom N1B). In contrast, the second serinol molecule adopts a more bent conformation: one hydroxyl group remains roughly coplanar with the carbon backbone, whereas the second hydroxyl group is oriented below the plane, in the same direction as the protonated amino group (Figure 6a, atoms N1A, O2A).

#### Comparative Conformational Analysis

A comparative analysis of the molecular conformation of serinol in the free base and its salts reveals clear trends in the spatial arrangement of the functional groups relative to the carbon backbone (Figure 7). In all structures, the observed conformations result from the interplay between directional hydrogen-bonding requirements and the optimization of crystal packing. The amino group consistently adopts a similar orientation in most structures, maintaining a relatively fixed position with respect to the carbon backbone (Figure 7a). In contrast, the hydroxyl groups display greater conformational variability, adjusting their orientation to maximize hydrogen-bonding interactions and to accommodate the geometry and coordination preferences imposed by the counterion. The N–C–C–O torsional angles (ψ_1_ and ψ_2_) for all derivatives are listed in Table 3. **SOx** and **SAd** exhibit nearly identical hydroxyl conformations for both hydroxyl groups in their serinol molecules, with ψ_1_/ψ_2_ values of 60.71(10)°/53.79(15)° for **SOx** and 65.2(3)°/–57.4(3)° for the first independent molecule of **SAd**. However, the second independent molecule of **SAd** adopts a markedly different conformation, with ψ_1_/ψ_2_ = 46.9(3)°/57.8(3)°, indicating a significant deviation in the hydroxyl orientation (see Figure 7b for the superposition of the two independent molecules). **SHCl** shows the most symmetric conformation of the series, with ψ_1_/ψ_2_ = 58.62(10)°/–58.01(12)°. This may be related to the reduced steric demand of accommodating a chloride anion in the packing arrangement, allowing both hydroxyl groups to adopt similar orientations. **SFB** and **SSt** differ significantly from the other derivatives in at least one of the two hydroxyl orientations. In **SFB**, the two crystallographically independent molecules have ψ_1_/ψ_2_ = 177.80(12)°/–64.39(14)° and −170.96(12)°/68.48(15)°, respectively, indicating a markedly different orientation of one hydroxyl group in each molecule, while **SSt** shows ψ_1_= −52.9(5)° and ψ_2_ = −57.5(5)°, the latter being similar to most of the other derivatives. This distinct ψ_1_value for **SSt** reflects the influence of specific intermolecular contacts with the stearate anion.

**Figure 7:**
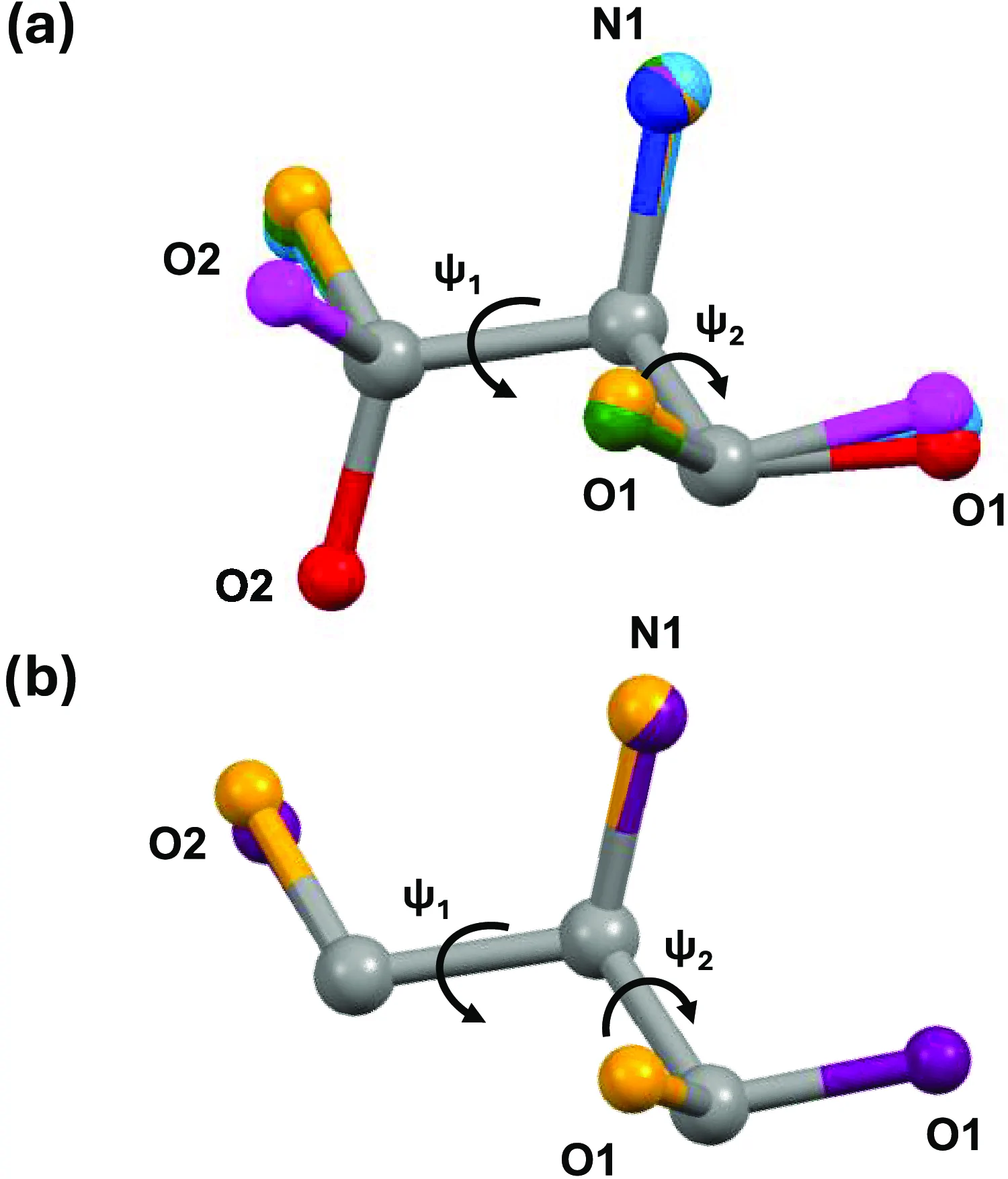
Comparison of the conformations of the serinol molecule in the crystal forms described in this paper. The graphical representation shows the different conformations of serinol in serinol free base and its salts. (a) Overlay of a serinol molecule for each compound with torsional angles ψ_1 and_ ψ_2_ highlighted with black arrows. Color code: carbon atoms, gray; **SFB**: N, blue, O, red; **SOx**: green; **SHCl**: light blue; **SSt**: pink; **SAd**: yellow. (b) Overlay of the two independent serinol molecules in the asymmetric unit of **SAd**. Color code: carbon atoms, gray; **SAd­(A)**: yellow; **SAd­(B)**: purple.

**Table 3:** N–C–C–O Torsional Angles (ψ, °) of the Serinol Molecule in the Free Base and Its Salts^a^

	ψ_1_	ψ_2_	ψ_1_	ψ_2_
**SFB**	177.80(12)	–64.39(14)	–170.96(12)	68.48(15)
**SOx**	53.79(15)	60.71(10)		
**SHCl**	58.62(10)	–58.01(12)		
**SSt**	–57.5(5)	–53.9(5)		
**SAd**	46.9(3)	57.8(3)	65.2(3)	–57.4(3)

a For structures containing two crystallographically independent serinol molecules (**SFB** and **SAd**), values are reported for both independent molecules.

Overall, this comparative evaluation highlights the decisive role of the HB contacts in the solid state in modulating the conformational preferences of serinol through tailored supramolecular interactions, ultimately influencing the overall crystal packing arrangement.

## Conclusions

This work reports the single-crystal structures of serinol, an important molecule from a pharmaceutical and industrial perspective, together with four of its salts: hemioxalate, hydrochloride, stearate, and adamantane-1-carboxylate. Crystals were obtained by slow-cooling crystallization from various alcoholic solvent mixtures and analyzed by single-crystal X-ray diffraction.

Across all structures, the crystal packing is dominated by an extensive network of hydrogen bonds, enabled by the hydroxyl and amino groups of serinol in concert with the functional groups of the respective counterions. In every salt studied, the structural and spectroscopic data are consistent with proton transfer from the coformer to the amino group of serinol. This behavior is consistent with the significant difference in p*K*_a_ values between the conjugate acids of the coformers and serinol itself, making proton transfer thermodynamically favorable in all cases. The specific molecular conformation adopted by serinol in each salt reflects an optimization of the hydrogen-bonding network within the crystal lattice, maximizing intermolecular stabilization.

This study represents a systematic structural exploration of serinol crystallization, providing fundamental insights into its solid-state behavior. These findings establish a structural framework that may guide future investigations aimed at tailoring its solid-state properties, potentially supporting the development of improved recovery, purification, and recycling approaches. By deepening the crystallographic understanding of serinol and its salts, this work contributes valuable knowledge that can inform the design of more sustainable and efficient processes in pharmaceutical and fine chemical production.

## Supplementary Material













## Notes

Crystallographic data of **SAd** (CIF)
Crystallographic data of **SFB** (CIF)
Crystallographic data of **SHCl** (CIF)
Crystallographic data of **SOx** (CIF)
Crystallographic data of **SSt** (CIF)
Crystallographic tables, additional images, and IR and NMR spectra (PDF)
