# Supplementary material for: Serinol in the Solid State: Structural Diversity and Packing Motifs in Free Base and Salt Forms
Source: ACS Omega. 2026 Aug 1;11(32):48681–91. doi: 10.1021/acsomega.6c06041 (PMC13491991; doi:10.1021/acsomega.6c06041)
Supplement: Supplementary file 1 [file ao6c06041_si_006.pdf]

Supporting Information

Serinol in the Solid State: Structural Diversity and Packing Motifs in Free Base and Salt Forms

Rachele Maschio,[a] Silvia Zampini,[b] Toni Grell,[b] Marco Vandone,[b] Fabio Travagin,[a] Luciano Lattuada,[c] Giovanni B. Giovenzana,[a] Valentina Colombo,*[b]*

[a] Dipartimento di Scienze del Farmaco, Università del Piemonte Orientale, Largo Donegani 2/3, 28100 Novara, Italy

[b] Dipartimento di Chimica, Università degli Studi di Milano, Via Golgi 19, 20133 Milano, Italy & UdR INSTM Milano

[c] Bracco Imaging SpA, Via Egidio Folli 50, 20134 Milano, Italy

KEYWORDS Serinol, Salts, Solid-state structure, X-ray diffraction, Aminodiol, Iopamidol

Table S1. List of the structures currently present in the Cambridge Structural Database (CSD) containing the serinol molecule.

CSD Refcode	Role of the serinol molecule
**AGIHAD**	Ligand in the preparation of a Ni substituted Krebs-type sandwich-tungstobismuthates, $\{(serinol)_2Ni_4(BiW_9)_2\}$, with antibacterial activiy.
**EXIFOJ**	Two protonated serinol molecules are part of the complex organic counter-cation $[Na_5(H_2O)_{18}\{(HOCH_2)_2CHNH_3\}_2]^{7+}$ which crystallizes with the Anderson–Evans type tungstoantimonate anion $[Sb^{V}W^{VI}{}_6O_{24}]^{7-}$. Serinol acts also as a mild buffering agent during the crystallization process.
**MAQWOU**	Four protonated serinol molecules coordinate six sodium ions to form the complex organic counter-cation $[Na_6((CH_2OH)_2CHNH_3)_4]^{10+}$ which counterbalance the paradodecatungstate B anion $[W12O40(OH)2]^{10-}$ in the $[Na_6(C_3H_{10}NO_2)_4][W_{12}O_{40}(OH)_2]\cdot 10H_2O$ compound.
**ORABUH**	Co-crystallization agent of the nonsteroidal-anti-inflammatory-drug flufenamic acid. The crystallization process leads to the formation of a simple organic salt which can form both hydro- and organo-gels.
**YAWLIU**	Co-crystallization agent of the nonsteroidal-anti-inflammatory-drug tolfenamic acid. The two compounds form a gelator salt which can form gels with chlorobenzene and bromobenzene.

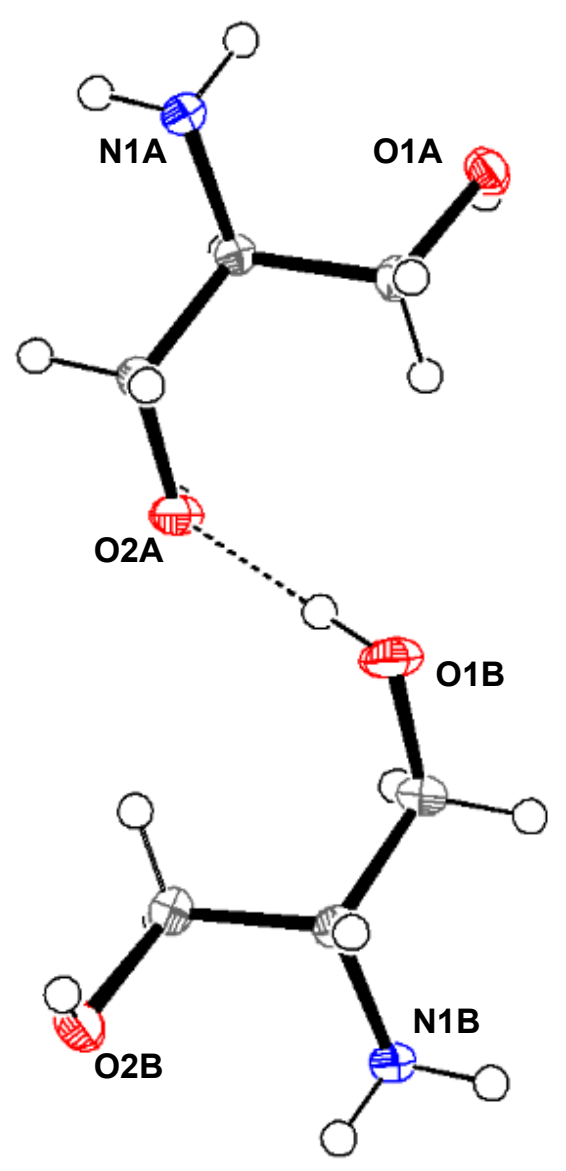

Figure S1. Ortep representations of **SFB** asymmetric units with labels on the heteroatoms. Color code: C, grey; N, blue; O, red; Cl, green; H, white. Ellipsoids are drawn at 50% probability.

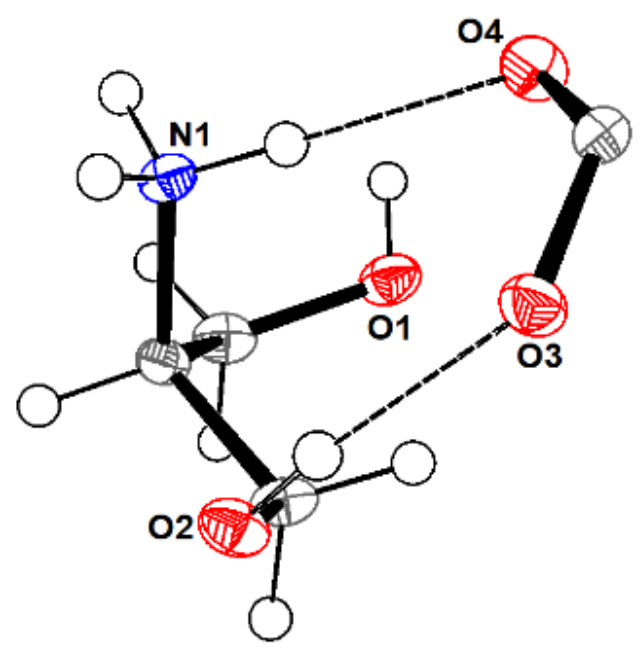

Figure S2. Ortep representations of **SOx** asymmetric units with labels on the heteroatoms. Color code: C, grey; N, blue; O, red; Cl, green; H, white. Ellipsoids are drawn at 50% probability.

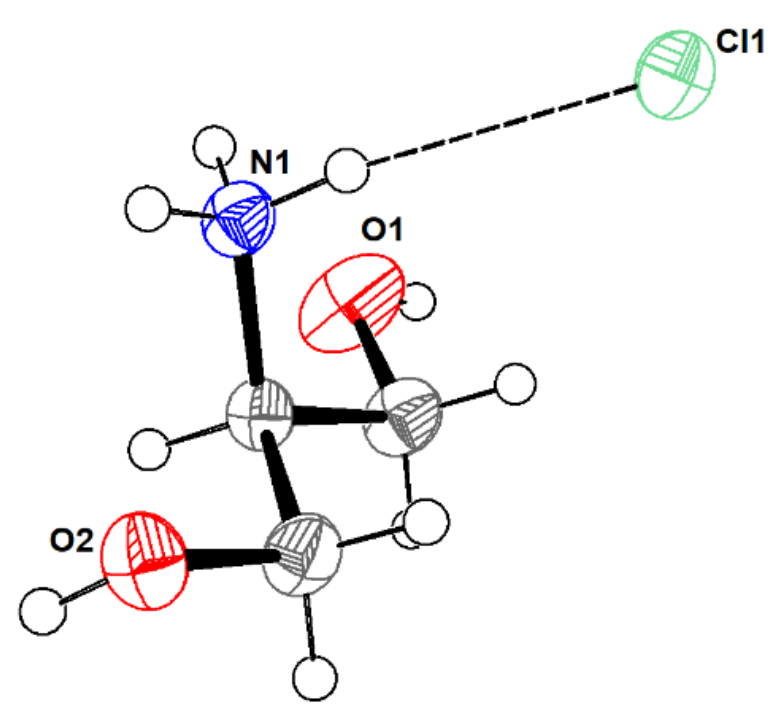

Figure S3. Ortep representations of **SHCl** asymmetric units with labels on the heteroatoms. Color code: C, grey; N, blue; O, red; Cl, green; H, white. Ellipsoids are drawn at 50% probability.

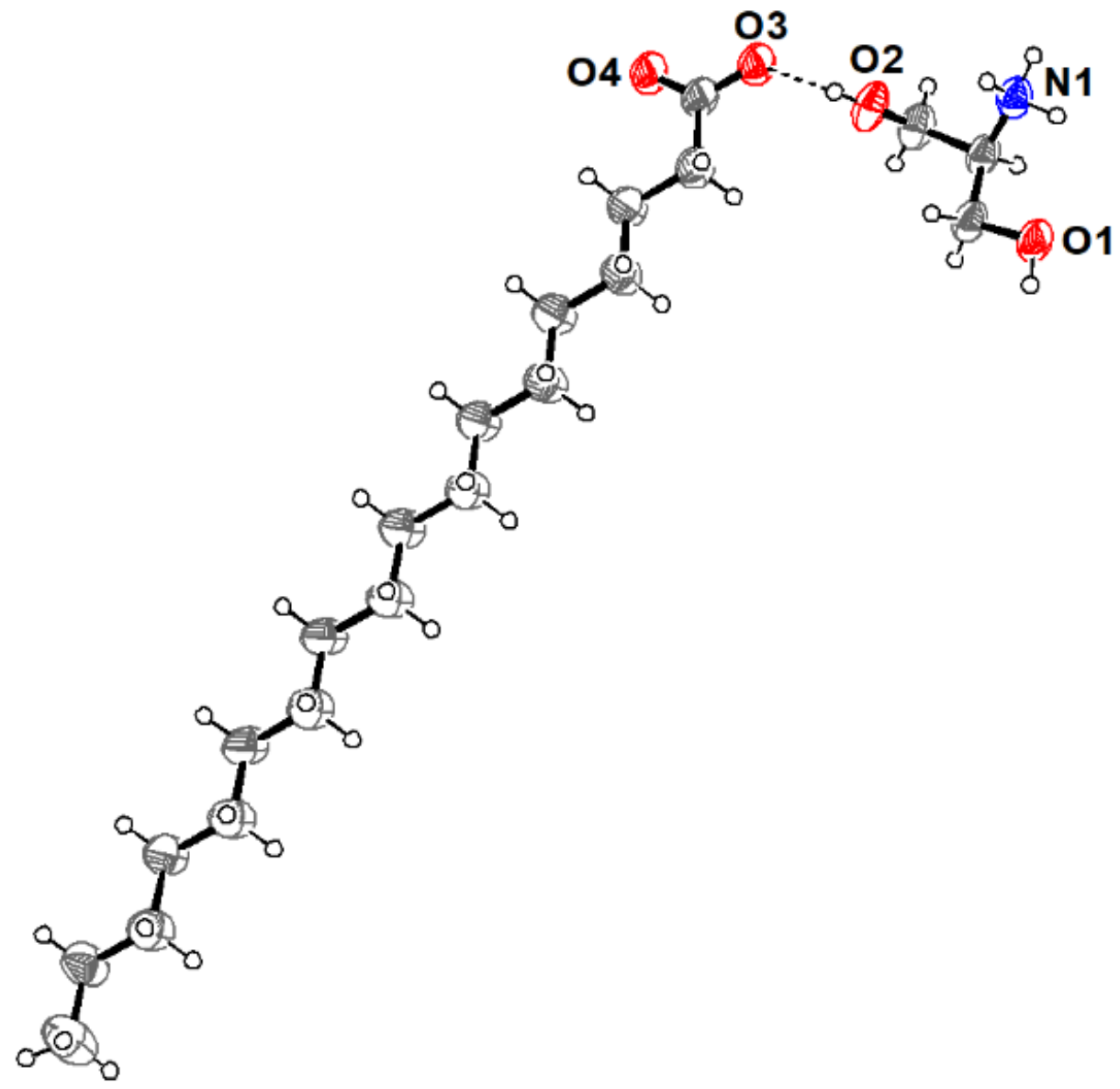

Figure S4. Ortep representations of **SSt** asymmetric units with labels on the heteroatoms. Color code: C, grey; N, blue; O, red; Cl, green; H, white. Ellipsoids are drawn at 50% probability.

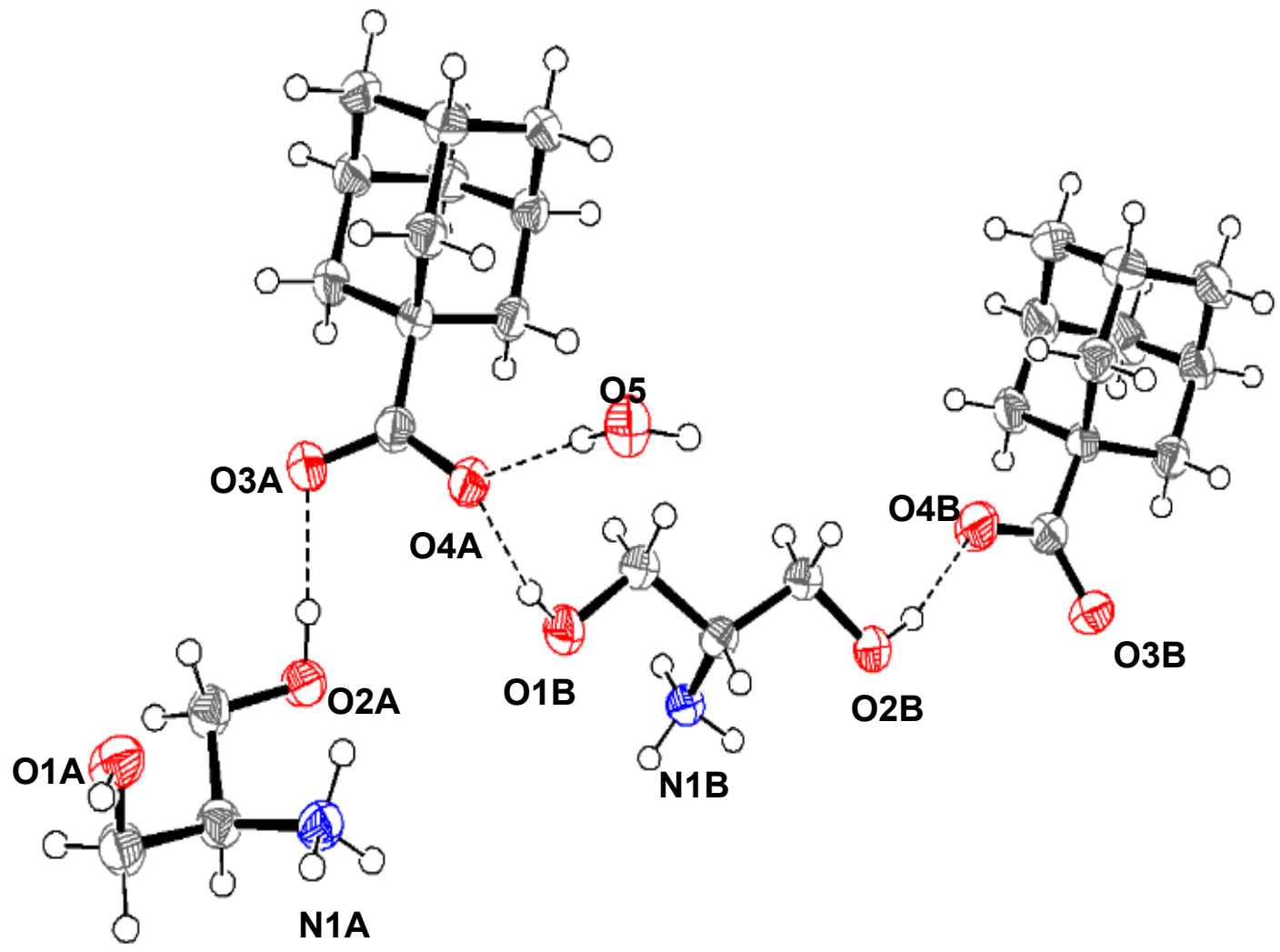

Figure S5. Ortep representations of **SAd** asymmetric units with labels on the heteroatoms. Color code: C, grey; N, blue; O, red; Cl, green; H, white. Ellipsoids are drawn at 50% probability.

Table S2. Distances [Å], angles [°] and type for hydrogen bond intermolecular interactions observed in SFB and in the salts discussed in this work.

Chemical compound	N°	Interaction type D-H···A	Distance / Å D(-H) ··· A	Angle / ° D-H-A
**SFB**	1	O1 -H7··· N1[1]	2.7697(17)	179(3)
	2	O2 -H8··· N2[2]	2.7321(17)	175(2)
	3	O3- H9 ··· O2	2.6601(17)	174(2)
	4	O4- H10··· O3[3]	2.7146(16)	170(2)
	5	N1- H11··· O1[4]	3.1446(19)	173(2)
	6	N1- H12··· O4[5]	3.1288(15)	158(2)
	7	N2- H13··· O4[6]	3.153(2)	174(2)
	8	N2- H14··· O1[7]	3.1239(15)	157(2)
[1]1/2-X,1/2+Y,1/2+Z; [2]1-X,2-Y,-1/2+Z; [3]1-X,1-Y,-1/2+Z; [4]+X,+Y,-1+Z; [5]-1/2+X,3/2-Y,+Z; [6]+X,+Y,1+Z; [7]1/2+X,3/2-Y,+Z				
**SOx**	1	O1- H1··· O2[3]	2.6867(7)	177.1
	2	O2- H2··· O3	2.6264(7)	172.2
	3	N1- H4··· O3[4]	2.8371(7)	166.5(10)
	4	N1- H4··· O4[5]	3.1025(8)	118.7(8)
	5	N1- H5··· O4	2.8006(8)	166.8(11)
	6	N1- H6··· O3[6]	3.0490(8)	153.0(10)
	7	N1- H6··· O4[1]	3.0107(8)	134.0(9)
[1]+X,-1+Y,+Z; [2]1/2-X,-1/2+Y,1/2-Z; [3]-1/2+X,1/2+Y,+Z; [4]-1/2+X,-1/2+Y,+Z; [5]1/2-X,3/2-Y,1-Z; [6]1-X,1-Y,1-Z				
**SHCl**	1	N1- H5··· Cl1	3.2935(9)	169.9(16)
	2	O2- H3··· Cl1[1]	3.2106(8)	163.3(18)
	3	N1- H4··· Cl1[2]	3.2634(8)	155.3(13)
	4	N1- H6··· O2[3]	2.9353(11)	148.2(17)
	5	O1- H1··· Cl1[4]	3.2204(9)	165.4(18)
[1]1/2+X,1/2-Y,1/2+Z; [2]+X,-1+Y,+Z; [3]1/2-X,-1/2+Y,5/2-Z; [4]1/2-X,-1/2+Y,3/2-Z				
**SSt**	1	O2- H4B··· O3	2.602(4)	177(6)
	2	N1- H4C··· O2[1]	2.791(5)	154(4)
	3	N1- H4D··· O1[2]	2.741(5)	143(4)
	4	N1- H4E··· O3[3]	2.701(5)	149(4)
	5	O1- H4A··· O4[4]	2.646(5)	166(4)
[1]-X,-Y,-Z; [2]-X,-1-Y,-Z; [3]-1-X,-Y,-Z; [4]1+X,-1+Y,+Z				
**SAd**	1	O1- H1 ··· O8[1]	2.725(3)	165.0
	2	O3- H3 ··· O8	2.754(3)	168.4
	3	O4- H4 ··· O6	2.615(3)	164.2
	4	N1- H2A··· O9[1]	2.822(3)	175(4)
	5	N1- H2B··· O1[2]	2.887(3)	173(4)
	6	N1- H2C··· O5[3]	2.739(3)	175(4)
	7	N2- H5A··· O3[4]	2.848(3)	149(3)
	8	N2- H5B··· O5[5]	2.726(3)	164(4)
	9	N2- H5C··· O2[4]	2.817(3)	157(4)
	10	O2- H6 ··· O7	2.675(3)	176(4)
	11	O9- H7 ··· O8	2.745(3)	168(4)
	12	O9- H8 O7[6]	2.762(3)	167(3)
[1]1-X,-1/2+Y,1/2-Z; [2]1-X,1/2+Y,1/2-Z; [3]1-X,1-Y,1-Z; [4]1-X,-Y,1-Z; [5]+X,-1+Y,+Z; [6]+X,1+Y,+Z				

Table S3. Torsional angles of SFB and its salts.

Chemical compound	Atoms<br>A B C D	Angle / °
**SFB**	C1 C2 C3 O2	-61.88(16)
	C4 C5 C6 O4	-172.42(11)
	N1 C2 C3 O2	177.80(12)
	N2 C5 C6 O4	68.48(15)
	O1 C1 C2 C3	176.21(11)
	O1 C1 C2 N1	-64.39(14)
	O3 C4 C5 C6	69.31(15)
	O3 C4 C5 N2	-170.96(12)
**SOx**	O1 C1 C2 N1	60.71(7)
	O1 C1 C2 C3	-59.82(7)
	N1 C2 C3 O2	53.69(7)
	C1 C2 C3 O2	175.19(5)
**SHCl**	O1 C1 C2 N1	-58.01(12)
	O1 C1 C2 C3	179.79(9)
	N1 C2 C3 O2	58.62(10)
	C1 C2 C3 O2	-179.39(8)
**SSt**	C1 C2 C3 O2	63.7(6)
	O1 C1 C2 C3	-173.5(4)
	O1 C1 C2 N1	-52.9(5)
	O3 C4 C5 C6	-176.9(4)
	O4 C4 C5 C6	3.7(7)
	N1 C2 C3 O2	-57.5(5)
**SAd**	C1 C2 C3 O2	168.4(2)
	C4 C5 C6 O4	179.5(2)
	N1 C2 C3 O2	46.9(3)
	N2 C5 C6 O4	-57.4(3)
	O1 C1 C2 C3	-63.9(3)
	O1 C1 C2 N1	57.8(3)
	O3 C4 C5 N2	-172.8(2)
	O3 C4 C5 N2	65.2(3)

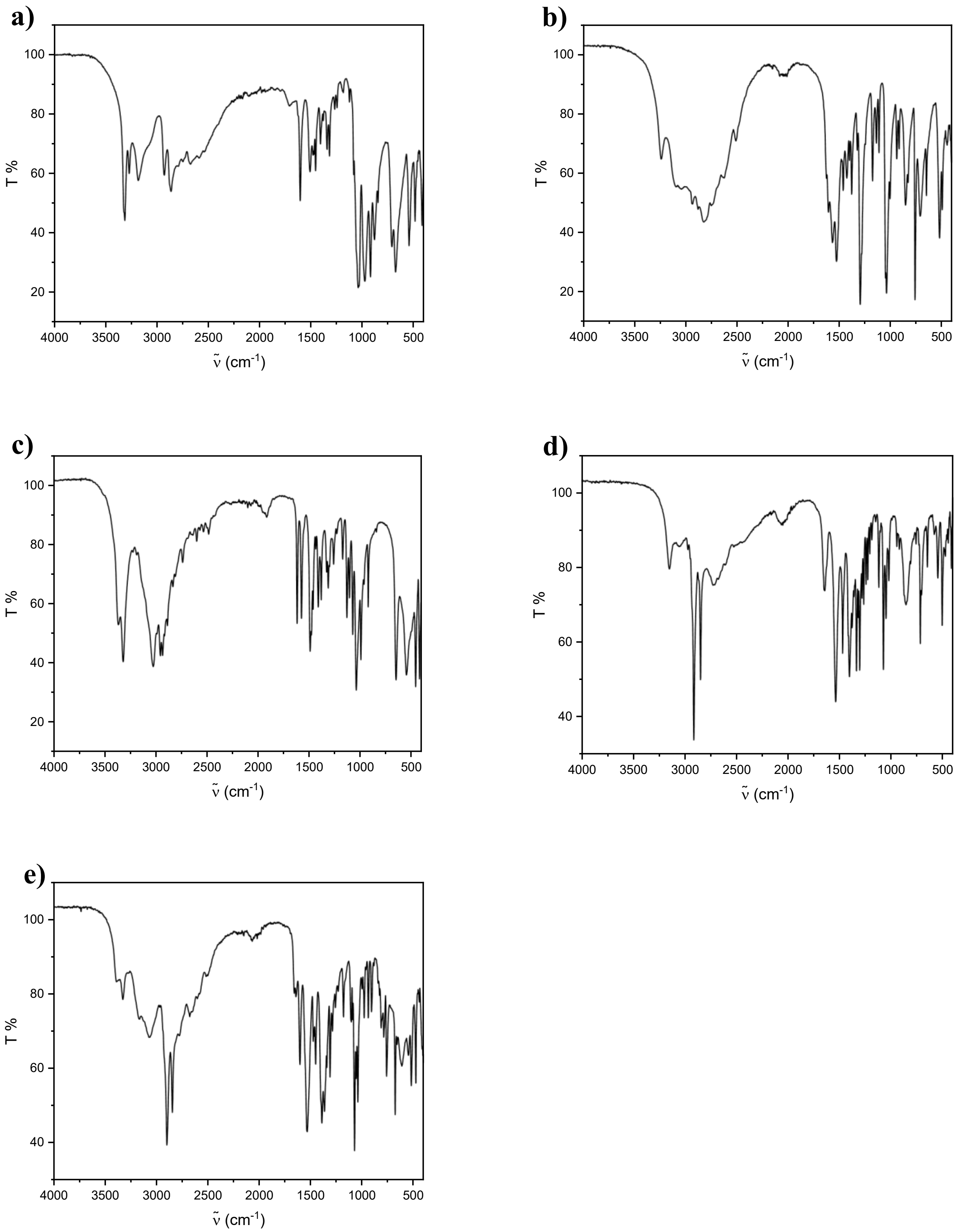

Figure S6. FT-IR spectra of (a) **SFB,** (b) **SOx**, (c) **SHCl**, (d) **SSt** and (e) **SAd**.

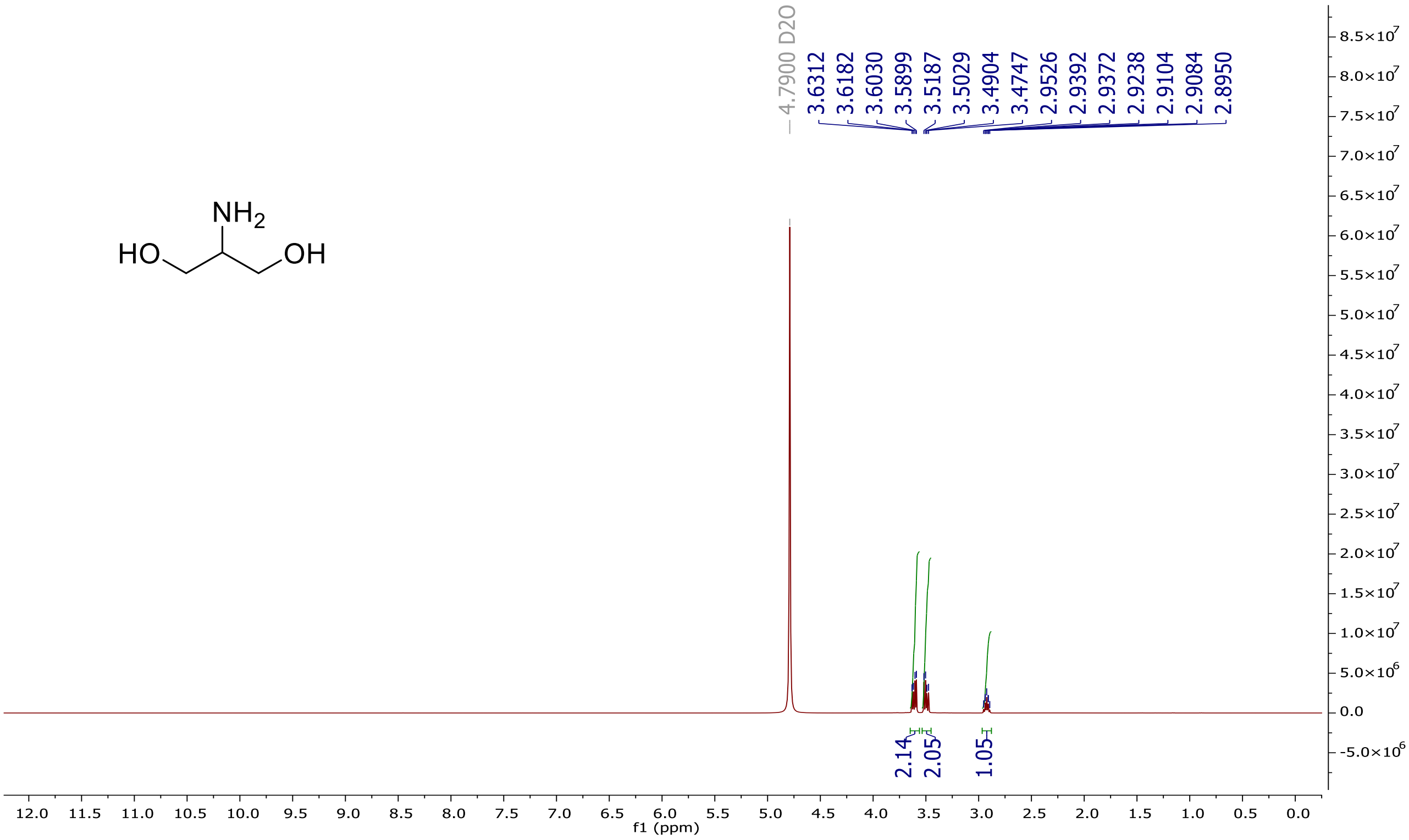

Figure S7. ^{1}H NMR s pectrum of ***SFB***.

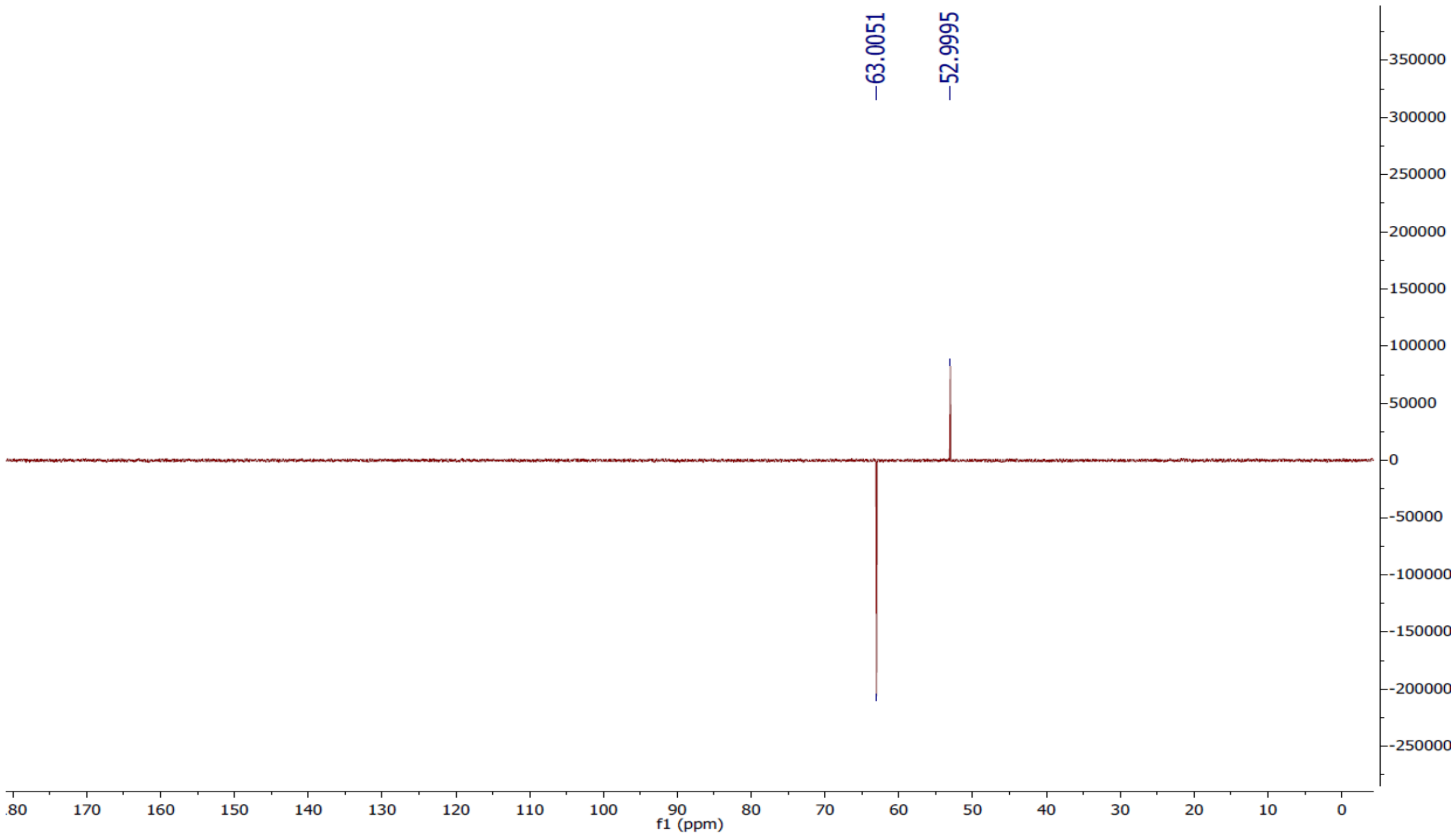

Figure S8. ^{13}C APT NMR spectrum of ***SFB***.

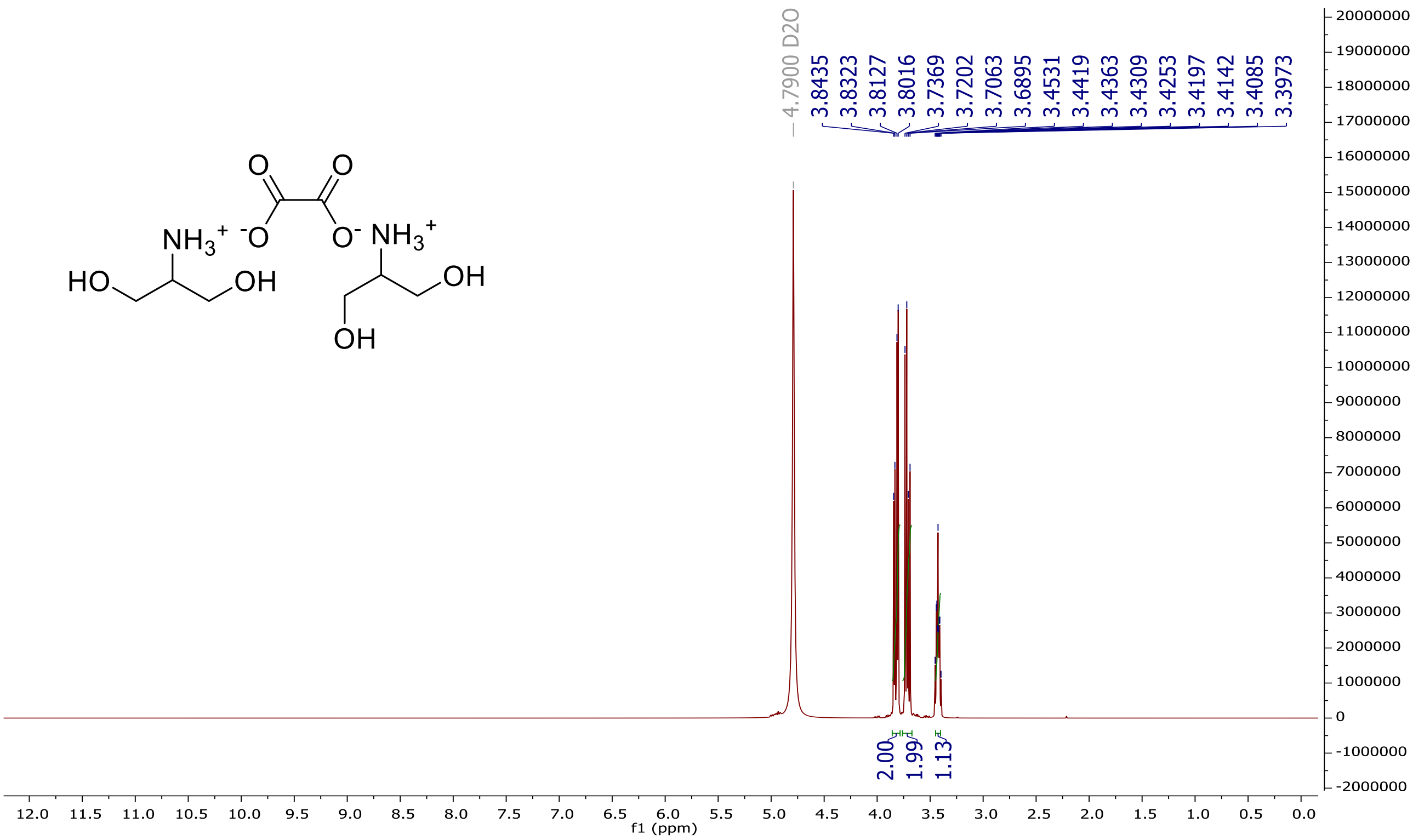

Figure S9. ^{1}H NMR spectrum of ***SOx***.

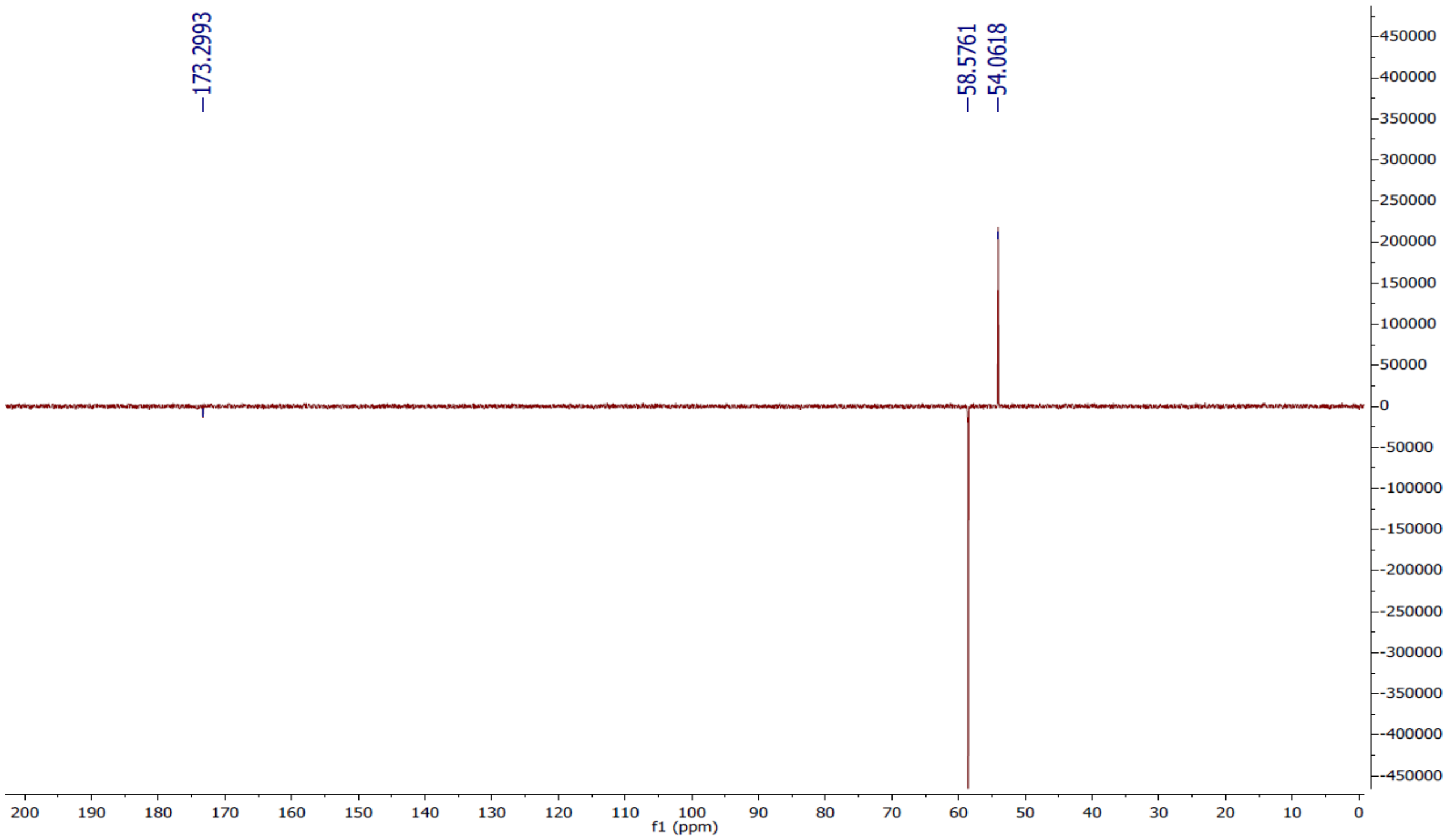

Figure S10. ^{13}C APT NMR spectrum of ***SOx***.

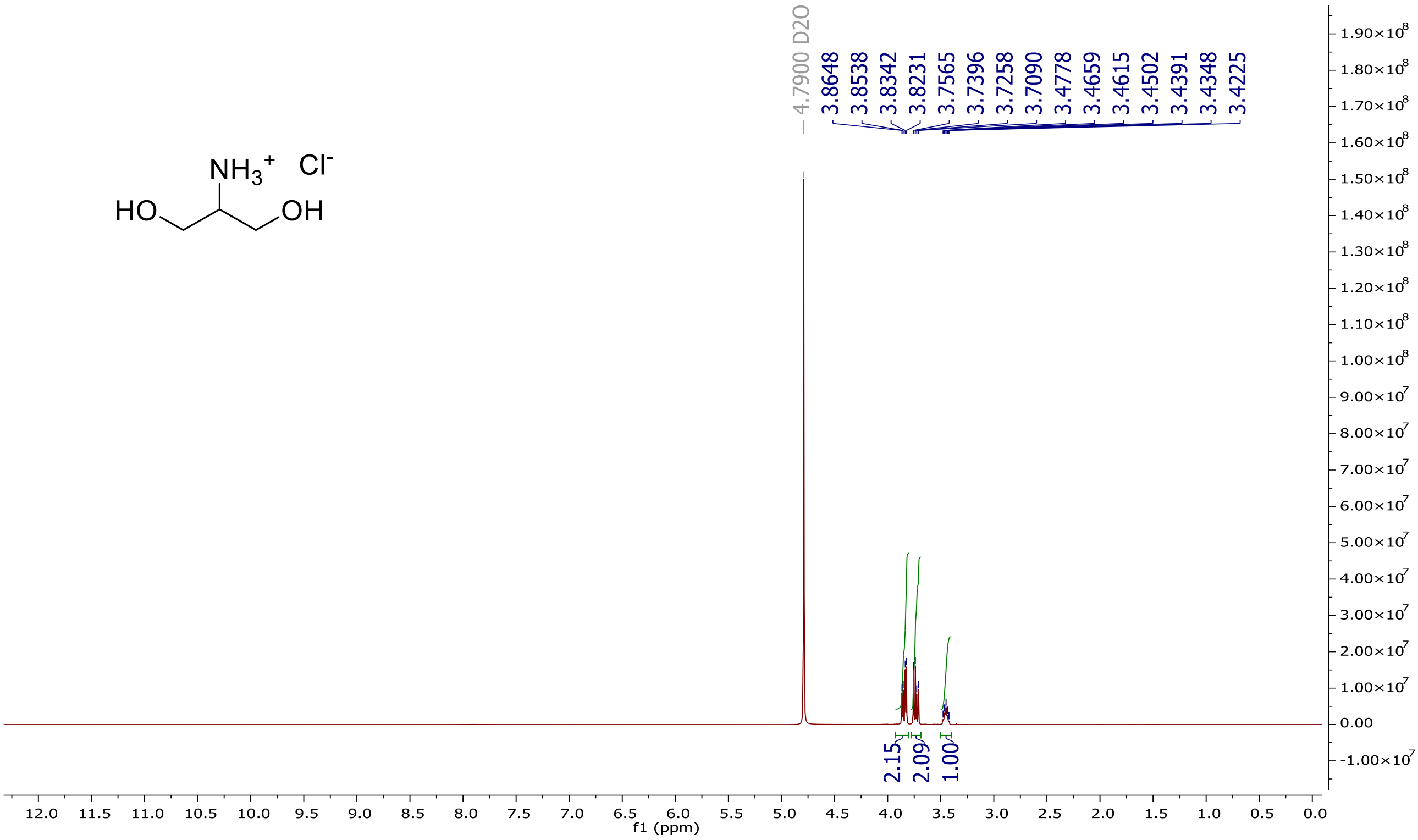

Figure S11. ^{1}H NMR spectrum of ***SHCl***.

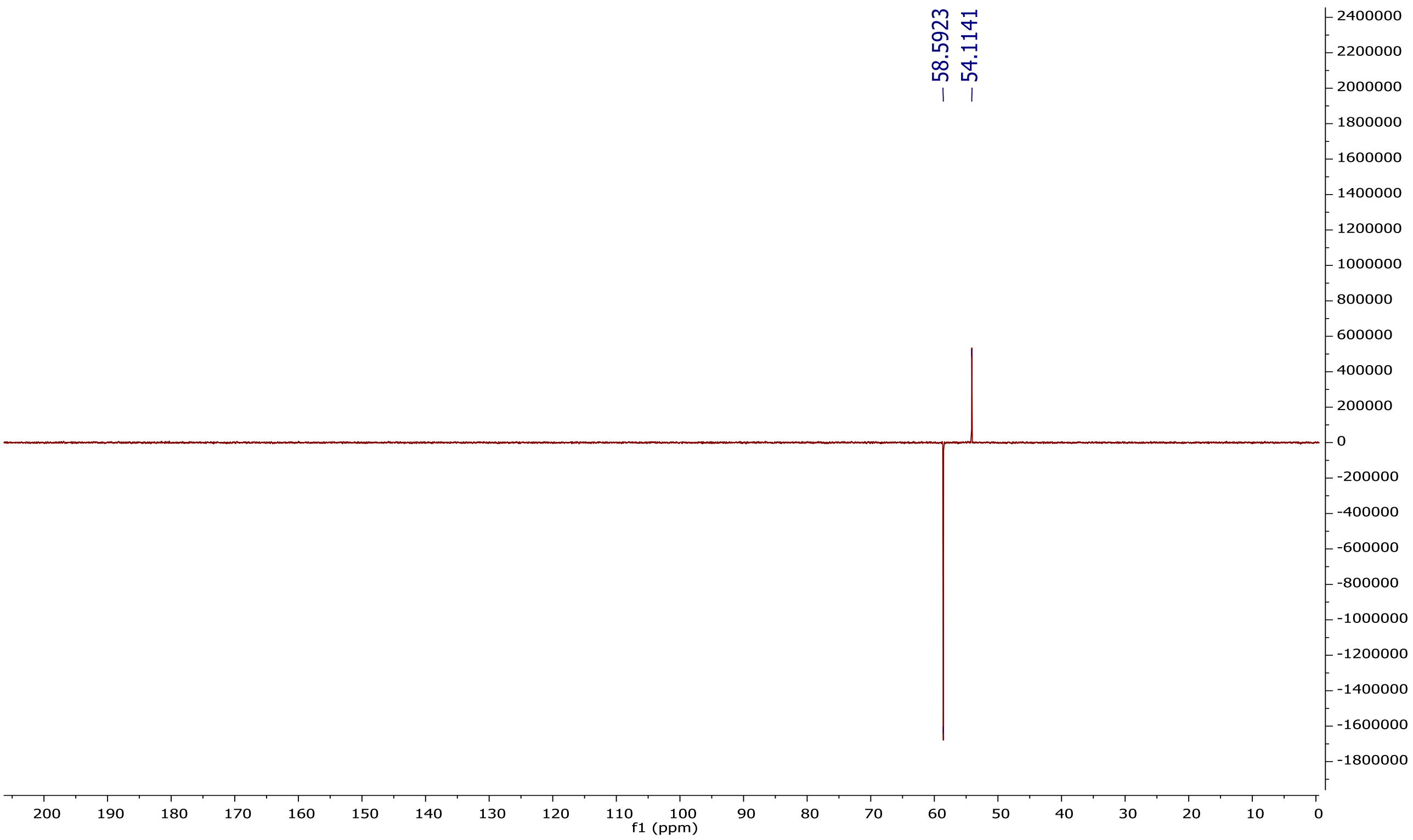

Figure S12. ^{13}C APT NMR spectrum of ***SHCl***.

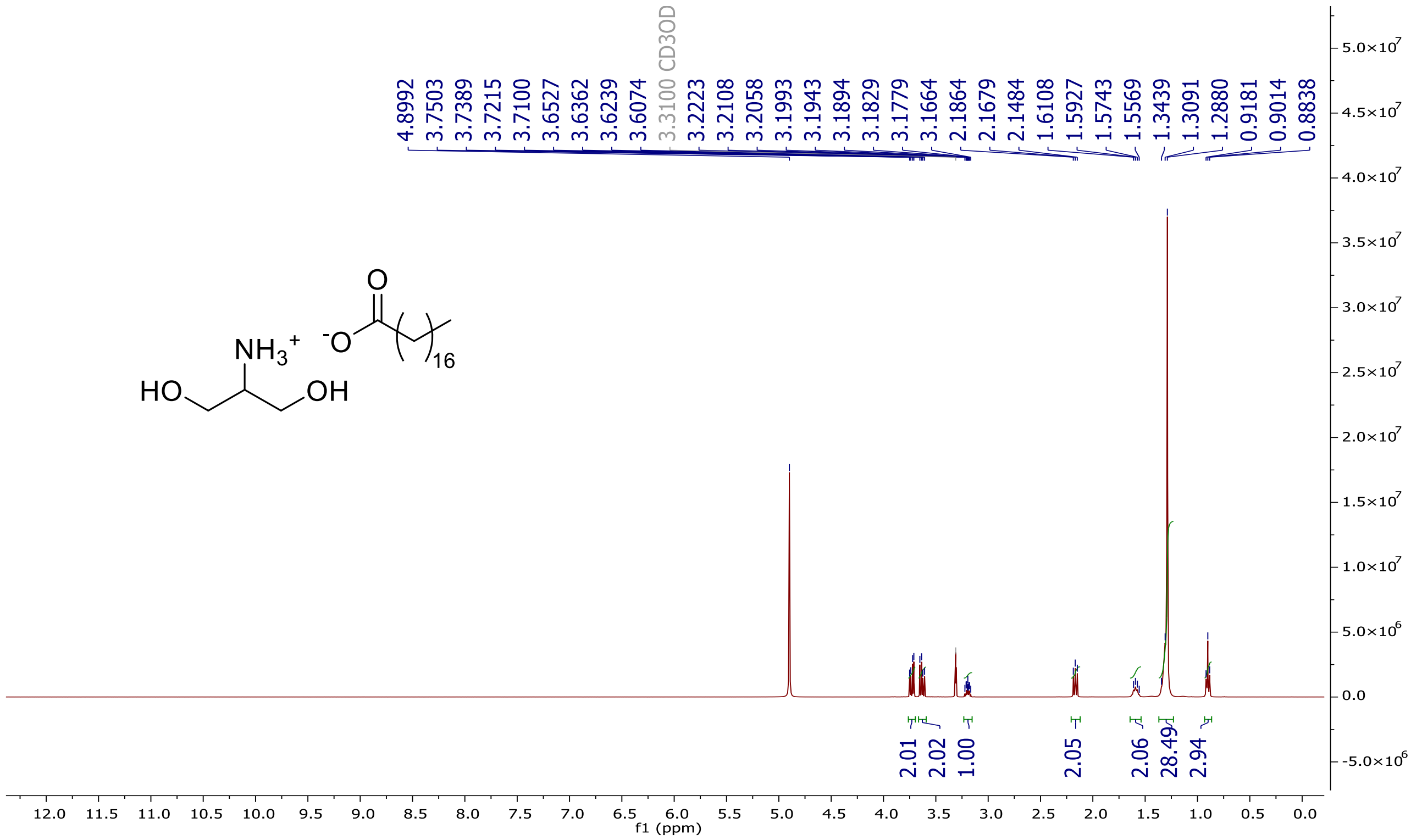

Figure S13. ^{1}H NMR spectrum of ***SSt***.

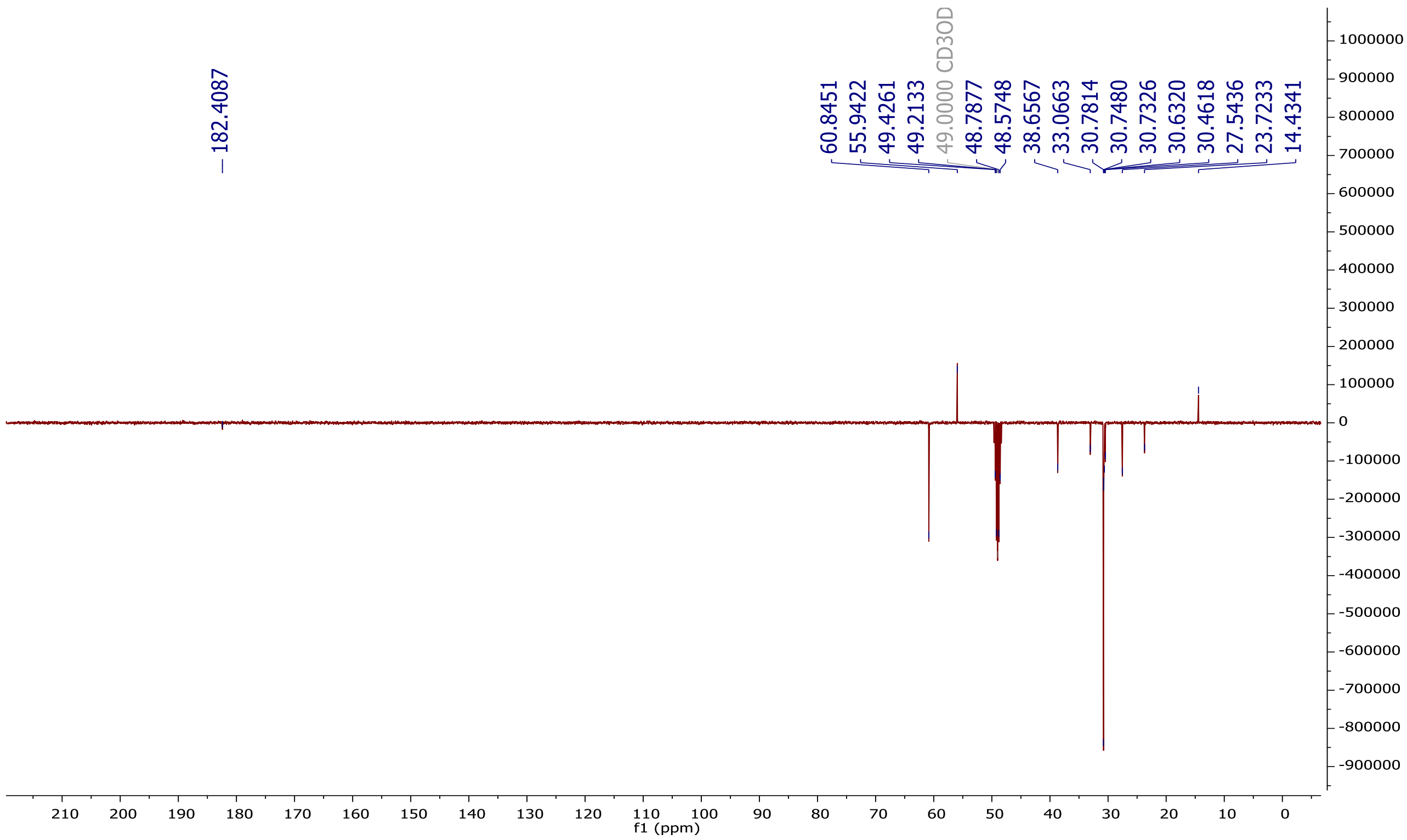

Figure S14. ^{13}C APT NMR spectrum of ***SSt***.

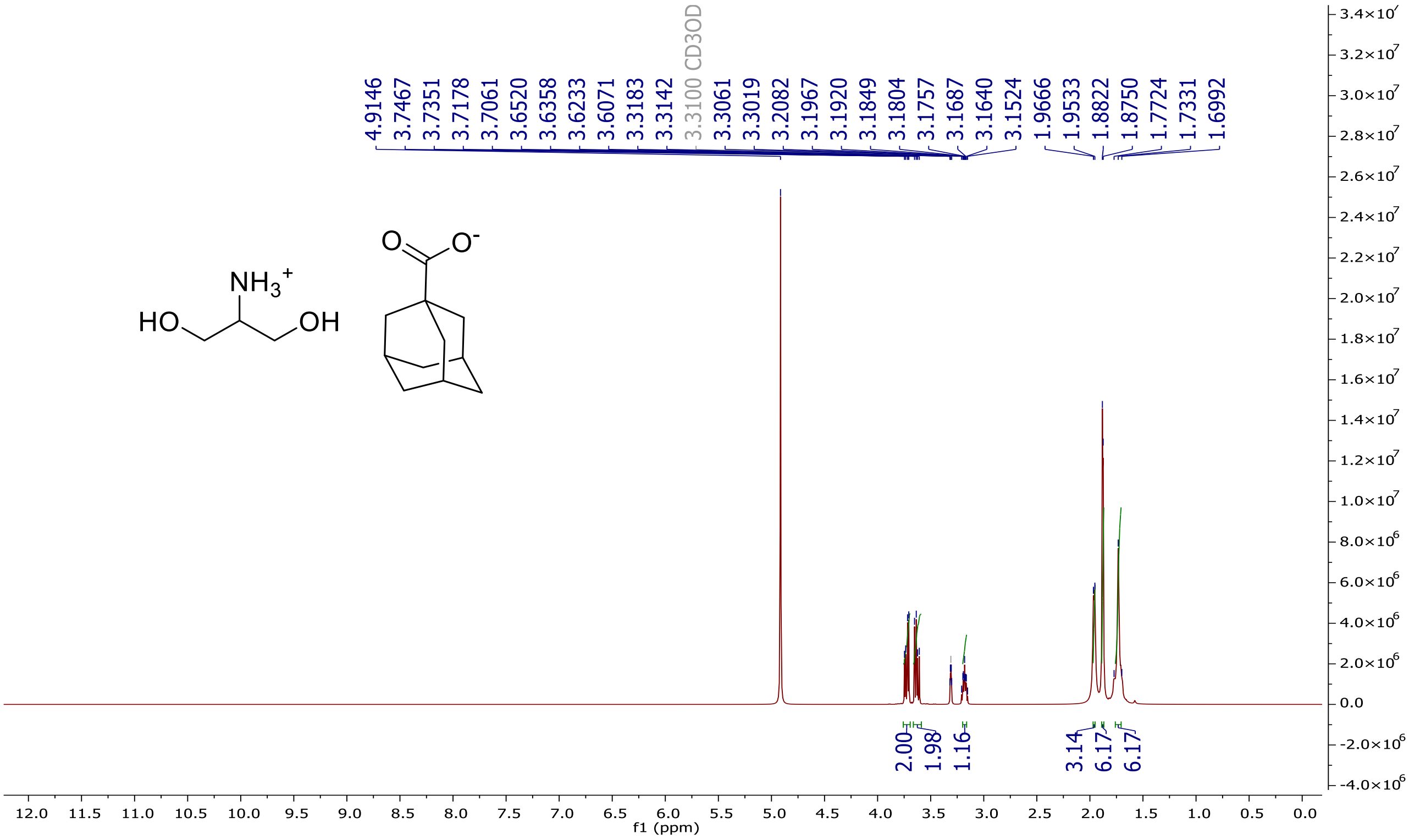

Figure S15. ^{1}H NMR spectrum of ***SAd***.

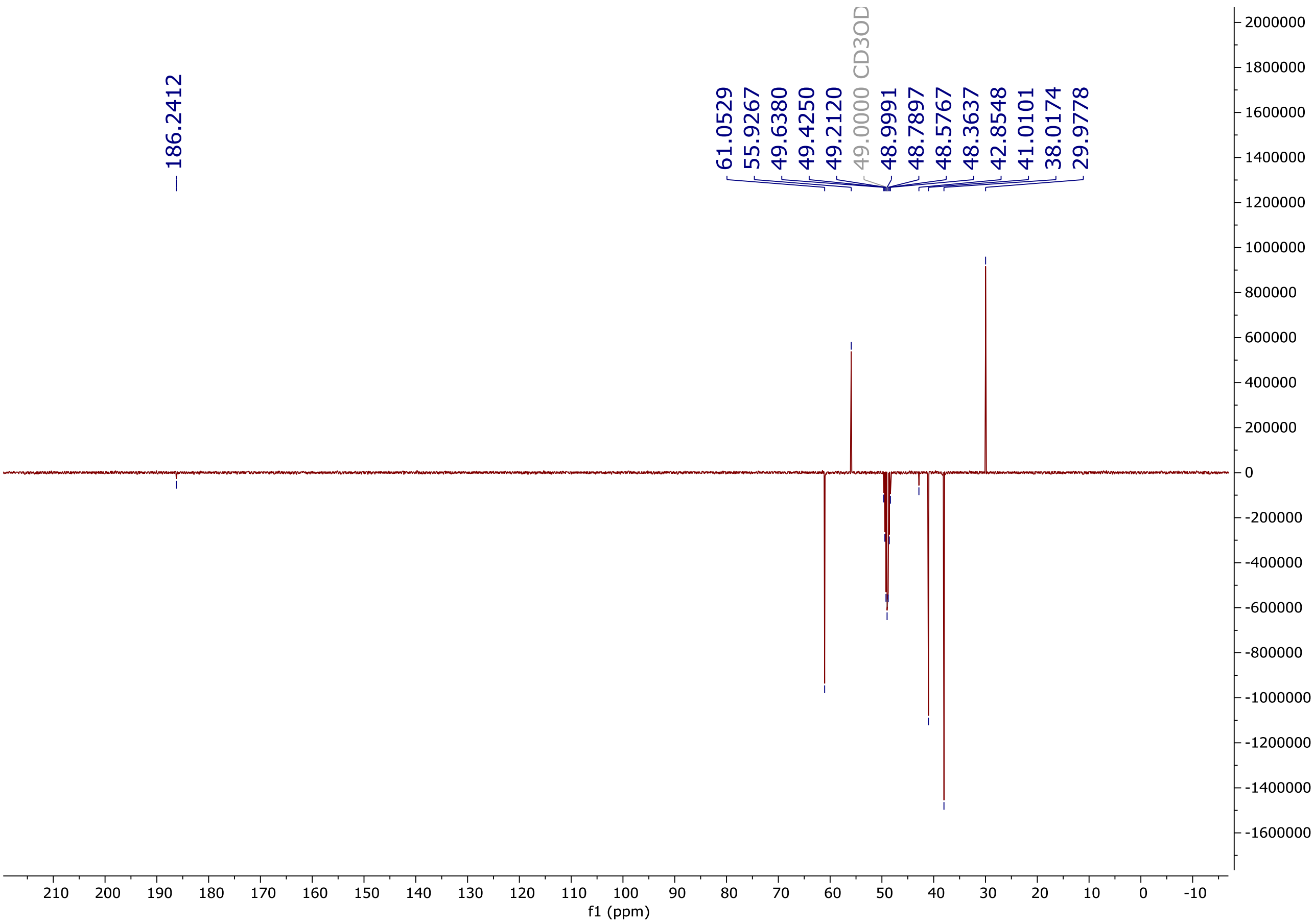

Figure S16. ^{13}C APT NMR spectrum of ***SAd***.
